# Lenvatinib plus transarterial chemoembolization and PD-1 inhibitors as conversion therapies for unresectable intermediate-advanced hepatocellular carcinoma: a phase 2 trial and exploratory biomolecular study

**DOI:** 10.1038/s41392-025-02498-z

**Published:** 2026-01-22

**Authors:** Xiaoyun Zhang, Haozheng Cai, Wei Peng, Haiqing Wang, JiaYi Wu, Xinrui Zhu, Weixin Guo, Fei Xie, Yu Zhang, Ming Wang, Yu Yu, Yongjie Zhou, Chuan Li, Junyi Shen, Chang Liu, Yu Yang, Xiaozhong Jiang, Qiu Li, Weixia Chen, Yujun Shi, Wusheng Lu, Xin Sun, Xielin Feng, Maolin Yan, Shuqun Cheng, Tianfu Wen

**Affiliations:** 1 Division of Liver Surgery, Department of General Surgery, West China Hospital, Sichuan University, Chengdu, China; 2 Chinese Evidence-based Medicine Center, West China Hospital, Sichuan University, Chengdu, China; 3 Department of Hepato-Biliary-Pancreatic Surgery, Sichuan Clinical Research Center for, Sichuan Cancer Hospital & Institute, Sichuan Cancer Center, Affiliated Cancer Hospital of University of Electronic Science and Technology of China, Chengdu, China; 4 The Shengli Clinical Medical College, Fujian Medical University, Fuzhou, China; 5 Department of Hepatobiliary Surgery, The First People’s Hospital of Changzhou, Changzhou, China; 6 Department of Hepatic Surgery VI, Eastern Hepatobiliary Surgery Hospital, Second Military Medical University, Shanghai, China; 7 Department of Hepatic-Biliary-Pancreatic Surgery, the First People’s Hospital of Neijiang, Neijiang, China; 8 Department of Hepatobiliary and Pancreatic Surgery, Sichuan Provincial People’s Hospital, University of Electronic Science and Technology of China, Chengdu, China; 9 Section for Hepatopancreatobiliary Surgery, Department of General Surgery, The Third People’s Hospital of Chengdu & The Affiliated Hospital of Southwest Jiaotong University, Chengdu, China; 10 Department of Hepatopancreatobiliary Surgery, the Second People’ s Hospital of Yibin, Yibin, China; 11 Laboratory of Liver Transplantation, Key Laboratory of Transplant Engineering and Immunology, NHC, West China Hospital, Sichuan University, Chengdu, China; 12 Department of Medical Oncology, West China Hospital, Sichuan University, Chengdu, China; 13 Department of Radiology, West China Hospital, Sichuan University, Chengdu, China; 14 Institute of clinical Pathology, Key Laboratory of Transplant Engineering and Immunology, NHC, West China Hospital, Sichuan University, Chengdu, China

**Keywords:** Gastrointestinal cancer, Gastrointestinal cancer, Outcomes research

## Abstract

Conversion therapy remains an uncommon strategy for managing unresectable hepatocellular carcinoma (uHCC) due to limited evidence supporting its efficacy. To address this gap, we initiated a prospective phase 2 multicenter trial (NCT04997850) comparing the LEN-TAP regimen, combining lenvatinib, transarterial chemoembolization (TACE), and PD-1 inhibitors, against TACE alone in uHCC patients. The study’s primary outcome was salvage liver resection (SLR) rate; secondary measures included objective response rate (ORR), overall survival (OS), event-free survival (EFS), recurrence-free survival (RFS), and safety profile. From October 2020 to November 2021, 142 eligible participants were assigned to LEN-TAP (n = 71) or TACE monotherapy (n = 71). At a median follow-up of 24.2 months, the LEN-TAP cohort exhibited a significantly higher SLR rate (59.2% vs. 18.3%, P < 0.001) and ORR (78.9% vs. 16.9%, P < 0.001). Median OS, EFS, and RFS were also substantially prolonged in the LEN-TAP cohort (not reached vs. 23.0 months, P < 0.001; 20.03 vs. 6.52 months, P < 0.001; 36.6 vs. 19.0 months, P = 0.048). Although grade 3 treatment-related AEs occurred more frequently with LEN-TAP (60.6% vs. 21.1%, P < 0.001), no grade 4 or higher toxicities were observed. Exploratory biomarker assessments via single-cell sequencing and flow cytometry linked elevated levels of circulating HLA-DR^+^CD38^+^CD8^+^ T cells with improved treatment response. These T cells appear to mediate antitumor activity potentially through the CXCR6–PI3K–AKT signaling axis. In summary, the LEN-TAP protocol demonstrates promising efficacy and acceptable tolerability as a conversion therapy in uHCC, with peripheral HLA-DR^+^CD38^+^CD8^+^ T cell abundance serving as a potential predictor of therapeutic benefit.

## Introduction

Hepatocellular carcinoma (HCC) remains a formidable global health challenge, ranking as one of the most commonly diagnosed malignancies and a leading cause of cancer-related death worldwide.^1^ A particularly alarming clinical reality is that almost half of all HCC patients are initially diagnosed with intermediate- or advanced-stage disease. At these stages, the tumor is often deemed unresectable due to factors such as multifocal bilobar involvement, macrovascular invasion, or inadequate future liver remnant, thereby severely limiting curative treatment options and portending a poor prognosis.^2^

Despite this, surgical resection with clear margins (R0) continues to offer the best opportunity for long-term survival and potential cure for eligible patients.^3,4^ This critical therapeutic gap between unresectable presentation and the curative potential of resection has given rise to the concept of “conversion therapy”. Conversion therapy for HCC is defined as a systemic and/or locoregional treatment strategy administered with the explicit intent of downstaging tumors, controlling disease progression, and ultimately converting initially unresectable HCC into resectable disease, enabling subsequent salvage liver resection (SLR).^5^ However, the adoption of conversion therapy in routine clinical practice has been hampered by a lack of robust, high-level evidence from prospective studies supporting its efficacy and safety.

For years, transarterial chemoembolization (TACE) has served as the cornerstone locoregional therapy for intermediate-stage HCC and has been explored as a monotherapeutic conversion strategy. However, its efficacy as a standalone conversion agent is modest, with reported conversion rates historically ranging from only 9.8% to 12%.^6^ This limitation is partly attributable to the compensatory upregulation of pro-angiogenic factors, such as vascular endothelial growth factor (VEGF) and fibroblast growth factor (FGF), induced by the post-TACE hypoxic microenvironment, which can paradoxically promote tumor revascularization, progression, and metastasis.^7^ The integration of molecular targeted therapy, specifically lenvatinib—a potent multi-kinase inhibitor targeting VEGF receptors (VEGFR1-3), FGF receptors (FGFR1-4), and other pathways—effectively counteracts this pro-angiogenic escape mechanism. Landmark trials, such as the LAUNCH study, have demonstrated that the combination of lenvatinib with TACE significantly improves objective response rates (ORR) and overall survival (OS) compared to lenvatinib monotherapy in advanced HCC, hinting at its substantial potential within a conversion framework.^8,9^

The introduction of immune checkpoint inhibitors (ICIs), particularly those directed against the programmed cell death-1 (PD-1) axis, has markedly transformed the therapeutic landscape in oncology. Recent studies indicate that TACE may augment the efficacy of immunotherapy through mechanisms such as triggering immunogenic cell death, liberating tumor-associated antigens, and reshaping the tumor immune microenvironment (TIME) to enhance T-cell infiltration and function.^10,11^ Encouraging outcomes, including improved response and conversion rates, have been observed in a number of retrospective analyses evaluating triple-combination regimens comprising TACE, a tyrosine kinase inhibitor (TKI; e.g., lenvatinib), and an anti-PD-1 agent in patients with unresectable hepatocellular carcinoma.^12–14^ However, the reliability of these findings is constrained by methodological limitations intrinsic to retrospective analyses, such as possible selection bias and restricted cohort sizes. The compelling biological rationale—whereby TACE provides localized tumor control and antigen release, lenvatinib normalizes tumor vasculature and suppresses immune-suppressive pathways, and PD-1 inhibitors reinvigorate pre-existing antitumor T-cell responses—suggests a potent synergistic effect worthy of investigation in a prospective trial setting.

In addition to evaluating clinical outcomes, identifying predictive biomarkers for treatment response is paramount for personalizing therapy and improving patient selection. Several studies across various malignancies have indicated that systemic immune activation, particularly the proliferation and functional restoration of CD8^+^ T cells in the peripheral blood, is associated with a favorable response to PD-1 inhibitor therapy.^15–18^ However, a specific and readily measurable immune subset predictive of response to the complex triple combination of lenvatinib, TACE, and PD-1 inhibitors (LEN-TAP) in HCC patients remains to be identified.

Therefore, we conducted this prospective, multicenter, phase 2 trial with a dual primary aim: first, to investigate the efficacy and safety of the sequential triple combination of lenvatinib, TACE, and PD-1 inhibitors versus TACE monotherapy as conversion therapy in patients with unresectable HCC; and second, through exploratory biomarker analysis, to identify a proliferating subset of CD8^+^ T cells whose dynamic changes in the peripheral blood may serve as a non-invasive biomarker for predicting therapeutic response.

## Results

### Patient characteristics

**Fig. 1:**
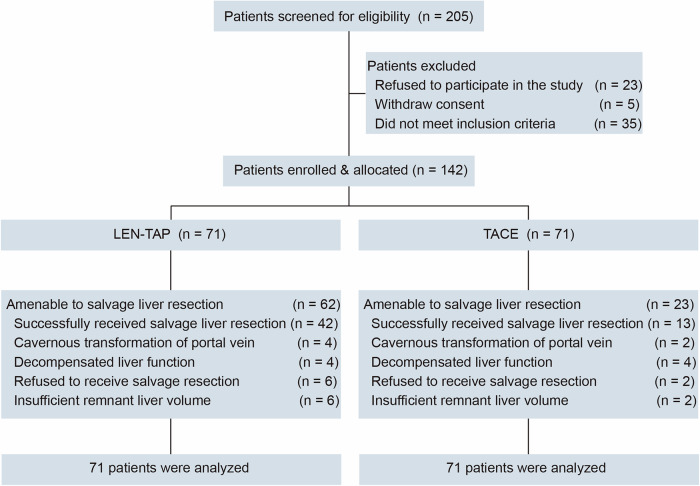
Patient Enrollment Flowchart. TACE, transarterial chemoembolization; LEN-TAP, lenvatinib-TACE–anti-PD-1 inhibitor combination therapy

Between October 1, 2020, and November 30, 2021, 205 consecutive patients were screened for eligibility, and 63 patients were excluded (Fig. 1). Overall, 142 (69%) of the 205 screened patients were deemed eligible and enrolled in this study. Seventy-one patients opted for LEN-TAP conversion therapy, and 71 patients chose TACE monotherapy as conversion therapy. One hundred thirty of the 142 patients (91.5%) were male. The most common underlying cause of HCC was hepatitis B virus infection (n = 116, 81.7%). All the participants had multinodular lesions (BCLC B stage) or macrovascular invasion (BCLC C stage): 43.0% had multinodular lesions, and 57.0% had portal vein or hepatic vein tumor thrombi. Except for the largest tumor size (11.1 ± 3.7 cm vs. 8.7 ± 4.1 cm, P < 0.001), the baseline characteristics were well matched between the cohorts (Table 1). The number of TACE sessions at the time of surgical resection or disease progression was 1.61 ± 0.85 in the LEN-TAP cohort and 2.63 ± 1.91 in the TACE cohort, with a significant difference between the two cohorts (P < 0.001). In addition, in the LEN-TAP cohort, the average number of PD-1 monoclonal antibody treatments received by patients at the time of surgical resection or disease progression was 7.72 ± 5.52.

**Table 1:** Baseline characteristics of enrolled patients

Characteristics	LEN-TAP group (n = 71)	TACE group (n = 71)	P value
Age, years, median (IQR)	53 (47–62)	57 (53–66)	0.131
Gender, n (%)			0.129
Male	68 (95.8)	62 (87.3)	
Female	3 (4.2)	9 (2.7)	
Etiology, n (%)			0.419
HBV	60 (84.5)	56 (78.9)	
HCV	1 (1.4)	3 (3.4)	
Others	10 (14.1)	12 (16.9)	
ECOG PS, n (%)			0.546
0	64 (90.1)	66 (93.0)	
1	7 (9.9)	5 (7.0)	
Child Pugh score, n (%)			0.262
5	63 (88.7)	58 (81.7)	
6	8 (11.3)	11 (15.5)	
7	0 (0)	2 (2.8)	
ALBI score	−2.63 ± 0.43	−2.65 ± 0.48	0.811
ALBI grade, n (%)			0.879
1	39 (54.9)	36 (50.7)	
2	31 (43.7)	34 (47.9)	
3	1 (1.4)	1 (1.7)	
BCLC stage, n (%)			0.865
B	30 (42.3)	31 (43.7)	
C	41 (57.7)	40 (56.3)	
Tumor number, n (%)			0.779
1	29 (40.8)	26 (36.6)	
2	28 (39.4)	19 (26.8)	
3	5 (7.0)	15 (21.1)	
≥4	9 (12.7)	11 (15.5)	
Largest tumor size, cm	11.1 ± 3.7	8.7 ± 4.1	<0.001
Vascular invasion, n (%)			0.818
Both PVTT and HVTT	6 (8.5)	4 (5.6)	0.718
HVTT	3 (4.2)	5 (7.0)	
PVTT	32 (45.2)	31 (43.7)	
VP1/VP2/VP3/VP4	5/6/19/8	2/7/19/7	
AFP level, n (%)			0.499
< 400 ng/mL	38 (53.5)	31 (59.2)	
≥ 400 ng/mL	33 (46.5)	40 (40.8)	

*IQR* Inter quartile range, *HBV* hepatitis B virus, *HCV* hepatitis C virus, *ECOG PS* eastern cooperative oncology group physical status, *ALBI* albumin-bilirubin, *BCLC* Barcelona clinic liver cancer, *PVTT* portal vein tumor thrombus, *HVTT* hepatic vein tumor thrombus, *AFP* alpha-fetoprotein, *TACE* transarterial chemoembolization, *LEN-TAP* triple combination regimen of Lenvatinib, transarterial chemoembolization (TACE) and PD-1 inhibitors

### Efficacy of LEN-TAP conversion therapy

A total of 62 (87.3%) patients in the LEN-TAP cohort and 23 (35.2%) in the TACE cohort met the criteria for SLR (Fig. 1). The remaining 57 patients (9 in the LEN-TAP cohort and 48 in the TACE cohort) were not considered eligible for SLR for the following reasons: disease progression (n = 42), ICG-R15 > 20% (n = 9), and poor physical status (n = 6). Ultimately, a total of 42 (59.2%) patients in the LEN-TAP cohort and 13 (18.3%) patients in the TACE cohort were successfully treated with SLR (P < 0.001).

**Fig. 2:**
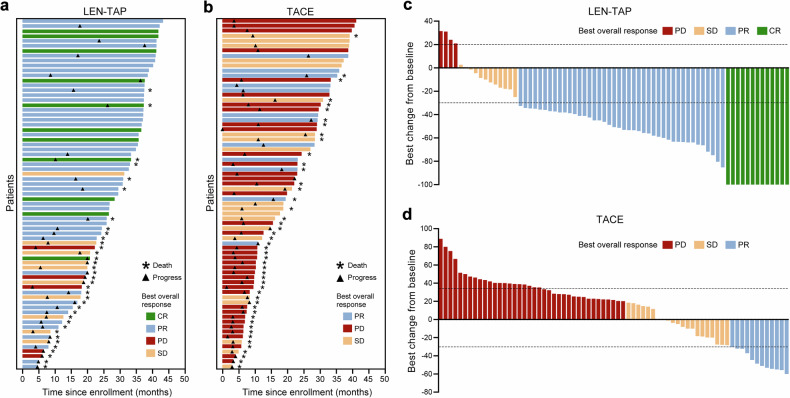
Treatment Efficacy. **a** Swimmer plot of overall survival for the LEN-TAP cohort (n = 71). Horizontal bars represent individual patients and are colored by best overall response (BOR): green (complete response, CR), blue (partial response, PR), yellow (stable disease, SD), and red (progressive disease, PD). Bar length indicates time from enrollment to last follow-up or death. Asterisks and triangles denote death and progression events, respectively. **b** Corresponding swimmer plot for the TACE cohort (n = 71). **c** Waterfall plot showing the maximum percentage change in target lesion diameter from baseline for the LEN-TAP cohort (n = 71), with colors corresponding to BOR. Pentagrams indicate patients with a reduction in target lesion diameter. **d** Waterfall plot for the TACE cohort (n = 71)

The tumor response rates and reduction rates are shown in Table 2 and Fig. 2. According to mRECIST, the ORR was 78.9% in 56/71 patients in the LEN-TAP cohort and 16.9% (12/71) in the TACE cohort (P < 0.001). In the RECSIST1.1, the ORR was 38.0% in the LEN-TAP cohort and 7.0% in the TACE cohort (P < 0.001). The disease control rate (percentage of patients achieving an objective response or SD) was 94.4% (66/71) in the LEN-TAP cohort and 43.7% (32/71) in the TACE cohort (P < 0.001) according to mRECIST, with corresponding values of 92.9% and 45.11% according to RECSIST 1.1 (P < 0.001). These data indicate that, compared with TACE monotherapy, LEN-TAP conversion therapy results in a superior tumor response and can also increase the percentage of uHCC patients who undergo salvage liver resection.

**Table 2:** Confirmed antitumor activity after conversion therapy

	RECIST 1.1			mRECIST		
Variable	LENTAP	TACE	P value	LENTAP	TACE	P value
Objective response	27 (38.0)	5 (7.0)	<0.001	56 (78.9)	12 (16.9)	<0.001
Complete response	1 (1.4)	0 (0)		13 (18.3)	0 (0)	
Partial response	26 (36.6)	5 (7.0)		43 (60.6)	12 (16.9)	
Stable disease	39 (54.9)	27 (38.0)		11 (15.5)	21 (29.6)	
Disease control	66 (92.9)	32 (45.1)	<0.001	67 (94.4)	33 (45.5)	<0.001
Progressive disease	5 (7.0)	39 (54.9)		4 (5.6)	38 (53.5)	
Amenable for salvage resection	58	21	<0.001	62	25	<0.001

*RECIST* response evaluation criteria in solid tumors, *mRECIST* modified RECIST

### Safety profile of LEN-TAP conversion therapy

AEs of any grade, regardless of causality, were more common in the LEN-TAP cohort (Table 3) and included hand–foot skin reactions (26.8% vs. 0, P < 0.001), diarrhea (32.4% vs. 15.5%, P = 0.018), vomiting (33.8% vs. 16.9%, P = 0.032), rash (21.1% vs. 0%, P < 0.001), hypertension (40.8% vs. 16.9%, P = 0.002), proteinuria (21.1% vs. 2.8%, P = 0.001), decreased PLT (35.2% vs. 14.1%, P = 0.003) and hypothyroidism (19.7% vs. 4.2%, P = 0.004). Grade 3 treatment-related adverse events (AEs) were more common in the LEN-TAP cohort (43/71, 60.6% vs. 15/71, 21.1%, P < 0.001), and no grade 4 or worse AEs were recorded. AEs leading to dose modification or interruption occurred in 12 (16.9%) patients who received LEN-TAP conversion therapy, i.e., 2 for abdominal pain, 1 for diarrhea, 4 for decreased platelet count, 3 for increased ALT, 1 for nausea and 1 for increased AST. Dose interruptions occurred in 14.1% (10/71) of patients (median duration 14 days, range 7–21), dose reductions occurred in 9.9% (7/71), and only 2.8% (2/71) required permanent discontinuation of lenvatinib (due to persistent Grade 3 decreased platelet count and diarrhea) while continuing other components. There were no new or unexpected toxicities; thus, LEN-TAP conversion therapy is safe.

**Table 3:** Adverse events after conversion therapy

Adverse events	Any grade, n (%)			Grade 3, n (%)			
	LENTAP (N = 71)	TACE (N = 71)	P	LENTAP (N = 71)	TACE (N = 71)	P	
Abdominal pain	42 (59.2)	34 (47.9)	0.178	10 (14.1)	2 (2.8)	0.012	
Hand-foot skin reaction	19 (26.8)	0 (0)	<0.001	0 (0)	0 (0)	NA	
Diarrhea	23 (32.4)	11 (15.5)	0.018	3 (4.2)	0 (0)	0.080	
Fatigue	27 (38.0)	25 (25.2)	0.782	1 (1.4)	1 (1.4)	1.000	
Nausea	42 (59.2)	34 (47.9)	0.178	6 (8.5)	0 (0)	0.012	
Vomiting	24 (33.8)	12 (16.9)	0.032	2 (2.8)	0 (0)	0.154	
Constipation	6 (8.3)	10 (14.1)	0.288	0 (0)	0 (0)	NA	
Decreased appetite	36 (50.7)	25 (35.3)	0.062	5 (7)	0 (0)	0.023	
Rash	15 (21.1)	0 (0)	<0.001	1 (1.4)	0 (0)	0.999	
Fever	24 (33.8)	21 (29.6)	0.588	4 (5.6)	0 (0)	0.042	
Hypertension	29 (40.8)	12 (16.9)	0.002	6 (8.5)	2 (2.8)	0.145	
Decreased weight	27 (38.0)	17 (23.9)	0.070	2 (2.8)	2 (2.8)	0.999	
Proteinuria	15 (21.1)	2 (2.8)	0.001	1 (1.4)	0 (0)	0.999	
Decreased platelets	25 (35.2)	10 (14.1)	0.003	8 (11.3)	1 (1.4)	0.049	
Increased ALT	38 (53.5)	33 (46.5)	0.401	10 (14.1)	2 (2.8)	0.016	
Increased AST	37 (52.1)	34 (47.9)	0.615	10 (14.1)	4 (5.6)	0.091	
Hyperbilirubinemia	12 (16.7)	14 (19.7)	0.509	0 (0)	1 (1.4)	0.999	
Hypothyroidism	14 (19.7)	3 (4.2)	0.004	1 (1.4)	0 (0)	0.999	

*ALT* alanine transaminase, *AST* aspartate aminotransferase

### Salvage liver resection after LEN-TAP conversion therapy

The median time from treatment initiation to salvage liver resection was 4.3 months in the LEN-TAP cohort and 2.8 months in the TACE cohort (P = 0.001). Laparoscopic surgery was performed in 4 patients (3 in the LEN-TAP cohort and 1 in the TACE cohort), while the remaining 51 patients underwent open surgery. Perioperative outcomes are summarized in Table 4. No significant differences were observed between the two cohorts in terms of complications, hospital stay, operative time, intraoperative blood loss, or transfusion requirement. Pathological assessment showed that significantly more patients in the LEN-TAP cohort achieved a major pathological response (MPR; 30 vs. 3, P = 0.003) or a pathological complete response (pCR; 17 vs. 0, P = 0.005) compared to the TACE cohort. Among patients with BCLC stage C disease, 21 in the LEN-TAP cohort successfully underwent SLR. Postoperative pathology revealed complete necrosis of the portal vein tumor thrombus in 10 (47.6%) of these patients, whereas viable tumor cells persisted in the thrombus in the remaining 11 (52.4%). In the TACE cohort, only 5 BCLC stage C patients underwent SLR, and all (100%) had residual tumor in the thrombus.

**Table 4:** Perioperative outcome for patients underwent salvage liver resection

Variables	LENTAP (n = 42)	TACE (n = 13)	P value
Hospital stays after surgery (days)	9.17 ± 5.11	8.54 ± 3.66	0.683
Preoperative AFP, n (%)			0.756
Normal	25 (59.5)	7 (53.8)	
Elevated	17 (40.5)	6 (46.2)	
Extent of resection, n (%)			0.480
Major	33 (78.6)	9 (69.2)	
Minor	9 (21.4)	4 (30.8)	
Operation duration (min)	224.00 ± 96.09	211.54 ± 82.749	0.675
HIO (Min)	43.00 ± 24.25	26.77 ± 24.54	0.027
Intraoperative blood loss (mL)	514.29 ± 455.63	424.62 ± 429.197	0.533
Transfusion, n (%)			1.00
Yes	10 (23.8)	3 (23.1)	
No	32 (76.2)	10 (76.9)	
Pathological response, n (%)			
pCR	17 (40.5)	0 (0)	0.005
MPR	30 (71.4)	3 (23.1)	0.003
Microvascular invasion, n (%)			0.427
Present	7 (16.7)	4 (30.8)	
Absent	35 (83.3)	9 (69.2)	
Clavien-Dindo classification, n (%)			0.664
0～Ⅱ	35 (83.3)	12 (92.3)	
Ⅲ～Ⅳ	7 (16.7)	1 (7.7)	

*AFP* alpha-fetoprotein, *HIO* hepatic inflow occlusion, *pCR* pathologic complete response, *MPR* major pathologic response

### Long-term outcomes of LEN-TAP conversion therapy

**Fig. 3:**
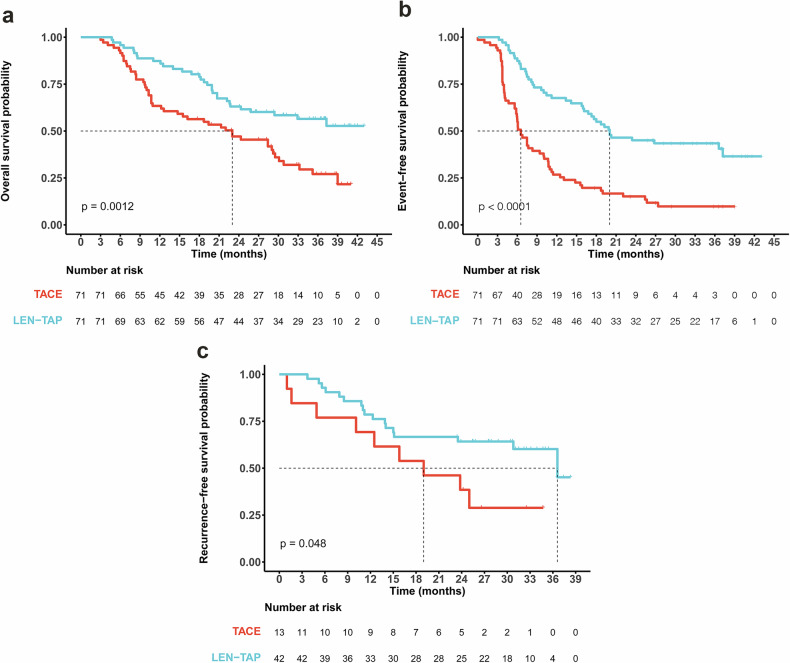
Kaplan–Meier Survival Analysis. Kaplan–Meier curves comparing (**a**) overall survival (OS), (**b**) event-free survival (EFS), and (**c**) recurrence-free survival (RFS) between the LEN-TAP and TACE cohorts. TACE, transarterial chemoembolization; LEN-TAP, lenvatinib plus TACE and anti-PD-1 inhibitors

According to the data cutoff date (January 31, 2024), the median duration of follow-up was 18.5 months (29.33 months in the LEN-TAP cohort and 19.83 months in the TACE cohort). A total of 31 (43.7%) patients in the LEN-TAP cohort and 48 (67.6%) patients in the TACE cohort died, and the median OS time was significantly longer with LEN-TAP than with TACE (NE vs. 23.0 months; hazard ratio, 0.48; 95% CI: 0.31--0.75; P < 0.001; Fig. 3a). A total of 42 (59.2%) patients in the LEN-TAP cohort and 63 (88.7%) patients in the TACE cohort experienced disease progression, death or recurrence after SLR (P < 0.001). The median EFS time was significantly longer with LEN-TAP than with TACE (20.03 months vs. 6.52 months; hazard ratio, 0.36; 95% CI: 0.24 to 0.54; P < 0.001; Fig. 3b). Seventeen patients in the LEN-TAP cohort and 9 patients in the TACE cohort experienced recurrence; the median RFS significantly differed between the two cohorts (36.6 vs. 19.0 months; hazard ratio, 0.45; 95% CI: 0.17--1.20; P = 0.048; Fig. 3c.

### Biomarker analysis

#### LEN-TAP conversion therapy leads to immune alterations, as assessed by single-cell sequencing

**Fig. 4:**
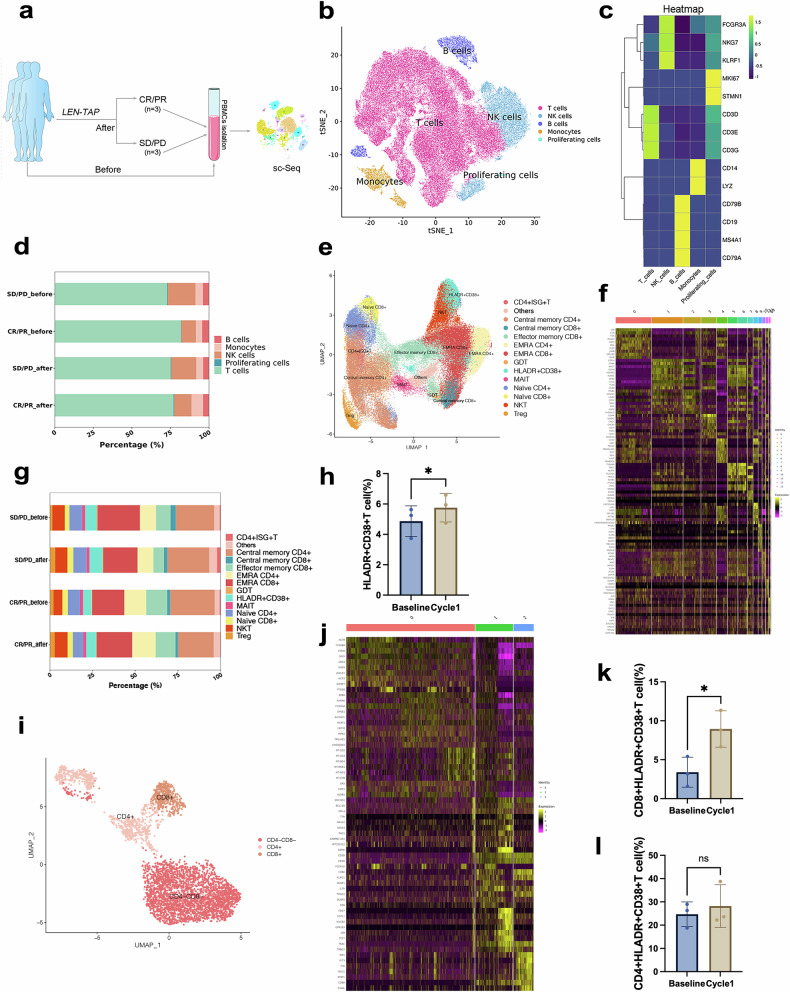
Immune alterations after LEN-TAP therapy were assessed via single-cell sequencing (sc-seq). **a** Schematic diagram of the PBMC single-cell transcriptome before and after LEN-TAP therapy. **b** t-distributed stochastic neighbor embedding (t-SNE) plot of the 5 identified main cell types in PBMCs. **c** Heatmap of feature signature genes in each cell type, where the colors from yellow to blue represent alterations from high expression to low expression. **d** Proportions of each cell type among PBMSCs in different groups. **e** UMAP representation of identified clusters in T cells. **f** Heatmap showing the top-ranked DEGs for each of the 13 T-cell clusters. **g** Proportions of each T-cell type in the different groups. **h** Distribution of HLADR^+^CD38^+^ T cells in PR/CR patients before and after treatment (∗ indicates P < 0.05). **i** UMAP plot of the 3 identified main cell types among the HLADR^+^CD38^+^ T cells (n= 3). **j** Log fold change (avg-logFC) gene expression of the top 10 marker genes for each cluster. **k** Distribution of HLA-DR + CD38 + CD8 + T cells in PR/CR patients before and after treatment (* indicates P < 0.05). **l** Distribution of CD4^+^HLA-DR^+^CD38^+^ T cells in PR/CR patients before and after treatment

As part of exploratory research, more readily available peripheral blood samples were collected and subjected to biomarker analysis. We performed single-cell sequencing of patients’ peripheral blood before and after LEN-TAP conversion therapy, which revealed changes in peripheral blood cell subpopulation ratios following conversion therapy compared with those before therapy. Peripheral blood mononuclear cells (PBMCs) derived from 6 patients (evenly clustered into the PR/CR and PD/SD cohorts) were freshly isolated and subjected to 10x Genomics single-cell sequencing (Fig. 4a). Ultimately, 100,367 PBMCs were obtained and sequenced. On the basis of their transcriptome data, these cells were clustered into five major cell types, namely, T cells, B cells, natural killer (NK) cells, monocytes and proliferating cells (Fig. 4b), and a heatmap of the top ranked signature genes and the proportions of each cell type are presented in Fig. 4c, d.

According to previous studies,^19^ T cells are the main effector cells that respond to PD-1 inhibitors. Therefore, we focused on alterations in T cells, which were further clustered into 13 cell types (Fig. 4e, f). A comparison of the proportions of these 13 clusters of T cells in PR/CR patients before and after treatment revealed that the proportions of HLADR^+^CD38^+^ T cells were most significantly increased after treatment (*P* = 0.039, Fig. 4g, h). T cells are divided into three types: CD4^+^ T cells, CD8^+^ T cells, and double-negative (CD4^-^CD8^-^) T cells. CD4^+^ T cells are mainly responsible for assisting in the activation and function of other immune cells, enhancing the efficacy of CD8^+^ T cells. CD8^+^ T cells, also known as cytotoxic T cells, are primarily responsible for identifying and killing cells infected with viruses or tumor mutations. However, double-negative (CD4^-^CD8^-^) T cells are relatively uncommon, and their specific role in the immune system remains a hot research topic.^20^ To further elucidate the underlying changes, the HLADR^+^CD38^+^ T cells were further divided into three subpopulations: single-positive CD4^+^ T cells, single-positive CD8^+^ T cells, and double-negative (CD4^-^CD8^-^) cells (Fig. 4i, j). Among these populations, only the HLA-DR^+^CD38^+^CD8^+^ T-cell population was significantly increased in PR/CR patients after treatment (*P* = 0.03, Fig. 4k, l). The DEGs in HLA-DR^+^CD38^+^CD8^+^ T cells before and after treatment and the related enrichment analysis results are presented in Supplementary Fig. S2.

### Validation in tumor tissues derived from uHCC patients via sc-Seq

**Fig. 5:**
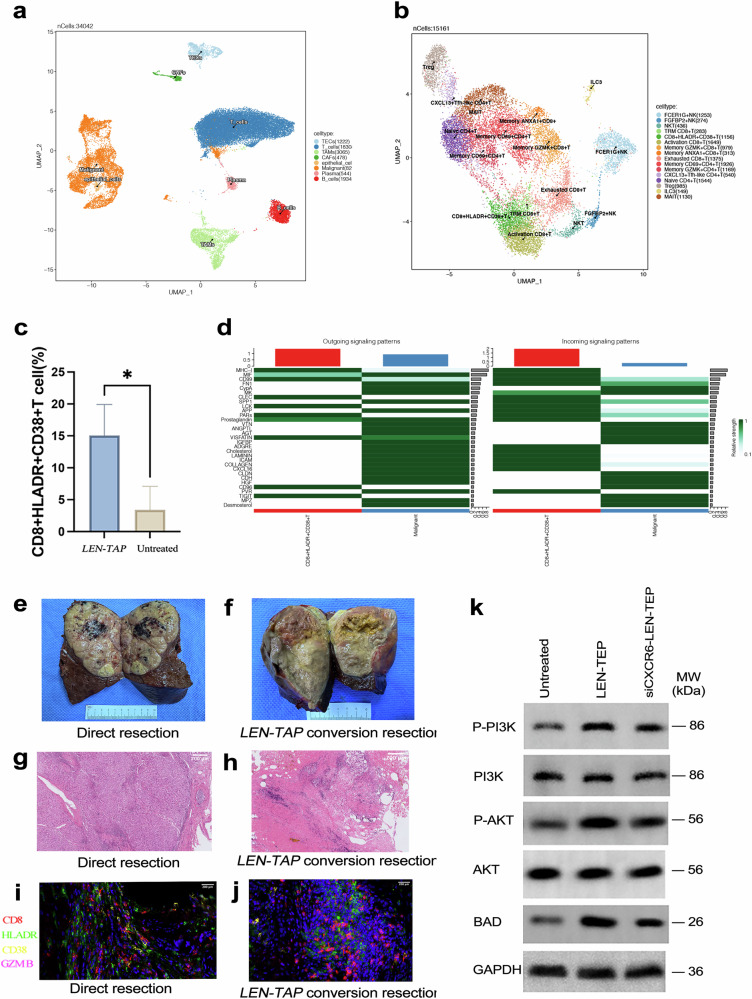
Changes in immune-related features induced by LEN-TAP therapy were assessed via single-cell sequencing (sc-seq) and in vitro experiments. **a** UMAP plot of the 8 identified main cell types. **b** UMAP representation of identified clusters in T cells. **c** Distribution of HLA-DR^+^CD38^+^ T cells in patients before and after treatment (∗ indicates P < 0.05). **d** Heatmap of the levels of the input and output signaling pathways. **e** Clinical samples of cancer tissues from patients who underwent direct resection. **f** Clinical samples of cancer tissues from patients who underwent conversion resection. **g** HE staining of specimens from patients who underwent direct resection. **h** HE staining of specimens from patients who underwent conversion resection. **i** Immunofluorescence analysis of HLA-DR^+^CD38^+^CD8^+^ T cells in biopsy samples obtained from HCC patients during direct resection. **j** Immunofluorescence analysis ofHLA-DR⁺CD38⁺CD8⁺ T cells in biopsy samples obtained from HCC patients during conversion resection. **k** Results of the detection of apoptosis-related proteins and PI3K–AKT pathway-related proteins after the coculture of each group of T cells with tumor cells

We performed single-cell RNA-seq on 34,042 liver cells from patients before and after LEN-TAP conversion therapy. Eight major cell types were identified—T cells, B cells, tumor-associated macrophages (TAMs), thymic epithelial cells (TECs), endothelial cells, plasma cells, cancer-associated fibroblasts (CAFs), and malignant tumor cells (Fig. 5a; Supplementary Fig. S3a–c). Focusing on T cells, we further identified 24 subtypes (Fig. 5b; Supplementary Fig. 3d, 3e) and observed a significant posttreatment increase in the proportion of HLA-DR⁺CD38⁺CD8⁺ T cells (P = 0.0018; Fig. 5c). The results of differential expression analysis between malignant cells and normal cells and pathway enrichment analysis of the DEGs are shown in Supplementary Fig. S3f–g.

CellChat analysis revealed 89 ligand–receptor networks between HLA-DR⁺CD38⁺CD8⁺ T cells and malignant cells—24 originating from T cells and 65 originating from tumor cells (Supplementary Fig. 2h). Malignant cells predominantly supply ligands, whereas HLA-DR⁺CD38⁺CD8⁺ T cells act mainly as receptors (Fig. 5d). Notably, CXCL16 (a ligand) from tumor cells and CXCR6 (a receptor) on HLA-DR⁺CD38⁺CD8⁺ T cells—previously implicated in enhancing anti-PD-1 therapy efficacy in other cancers—may mediate key tumor‒immune interactions.^21,22^

Immunofluorescence and H&E staining revealed that untreated HCC samples presented a disorganized architecture with sparse HLA-DR⁺CD38⁺CD8⁺ T cells, whereas therapy restored the tissue structure, reduced the tumor cell density, and promoted the infiltration of GZMB-expressing HLA-DR⁺CD38⁺CD8⁺ cells (Fig. 5e-j), suggesting enhanced cytotoxic activity. In functional assays with peripheral blood from five untreated patients and ten LEN-TAP–responsive patients (five used for CXCR6 knockdown), coculture for 72 h revealed that LEN-TAP treatment increased tumor-cell BAD expression; CXCR6 silencing attenuated this effect. KEGG pathway enrichment analysis suggested that the DEGs associated with HLA-DR⁺CD38⁺CD8⁺ T cells after conversion therapy were significantly enriched in the PI3K‒AKT signaling pathway. We further examined the expression levels of proteins related to the PI3K‒AKT signaling pathway in the three groups. Western blotting revealed elevated p-PI3K and p-AKT levels after LEN-TAP conversion therapy; these levels tended to decrease upon CXCR6 knockdown, whereas the total PI3K/AKT ratio remained unchanged (Fig. 5k).

### Increased HLA-DR^+^CD38^+^CD8^+^ T cells in the blood predict a better response to conversion therapy

**Fig. 6:**
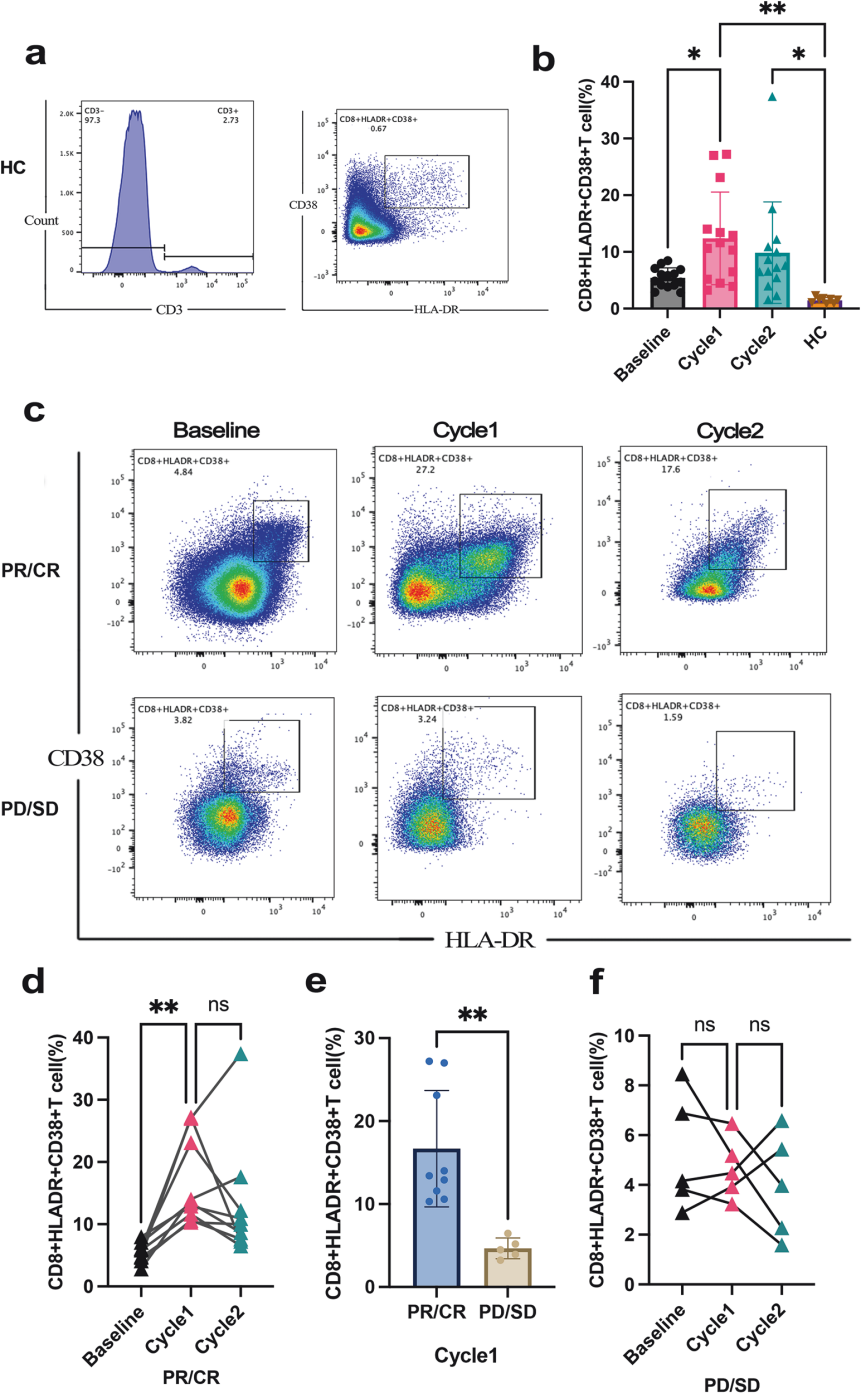
Flow Cytometric Analysis of T Cell Activation. **a** Proportion of CD3^+^HLA-DR^+^CD38^+^ T cells and CD3^+^HLA-DR^+^CD38^+^CD8^+^ T cells in the peripheral blood from healthy controls (HCs). **b** Distribution of CD3^+^HLA-DR^+^CD38^+^CD8^+^ T cells frequencies among different patient groups (*P < 0.05). **c** Dynamic changes in HLA-DR and CD38 co-expression on CD3^+^ T cells during the treatment course. **d** T Levels of CD3^+^HLA-DR^+^CD38^+^CD8^+^ T cells in patients who achieved a partial or complete response (PR/CR) (*P < 0.01). **e** Frequencies of CD3^+^HLA-DR^+^CD38^+^CD8^+^ T cells after the first cycle of immunotherapy, comparing responders (PR/CR) and non-responders (PD/SD) (*P < 0.01). **f** Distribution of CD3^+^HLA-DR^+^CD38^+^CD8^+^ T cells in patients with stable or progressive disease (PD/SD)

To validate the single-cell sequencing results, flow cytometry was used to identify changes in immune cell subsets in patient blood samples. Significant differences in peripheral blood HLA-DR^+^CD38^+^CD8^+^ T cells were observed between healthy controls (HCs) and patients before treatment, at the first dose of PD-1 inhibitor (cycle 1), and at the second dose of PD-1 inhibitor (cycle 2) (Fig. 6a, b; F = 5.942, P = 0.0016). The percentage of HLA-DR^+^CD38^+^CD8^+^ T cells in HCs was much lower than that in treated patients (Fig. 6b HCs vs. cycle 1, 1.45 ± 0.51% vs. 12.39 ± 8.15%, *P* = 0.0024; HCs vs. cycle 2, 1.45 ± 0.51% vs. 9.86 ± 8.95%, *P* = 0.03). Compared with patients who were not treated, patients who received the first dose of PD-1 inhibitors had a greater percentage of HLA-DR^+^CD38^+^CD8^+^ T cells (12.39 ± 8.15% vs. 9.86 ± 8.95%, *P* = 0.03; Fig. 6b).

We subsequently analyzed the changes in HLA-DR^+^CD38^+^CD8^+^ T cells between PR/CR patients and PD/SD patients during treatment. The number of HLA-DR^+^CD38^+^CD8^+^ T cells significantly increased during the clinical treatment of PR/CR patients (Fig. 6c, d, F = 7.003, *P* = 0.0059), and this alteration was greater in PR/CR patients receiving the first dose of PD-1 inhibitors than in untreated PR/CR patients (Fig. 6d, P = 0.0037, 5.50 ± 1.57% vs. 16.68 ± 7.02%). In addition, the proportion of these cells in the blood was greater in PR/CR patients than in PD/SD patients after the first dose of PD-1 inhibitor (Fig. 6e, P = 0.0029, 16.68 ± 7.02% vs. 4.66 ± 1.24%). However, no significant differences in these cells during the treatment of PD patients were found (Fig. 6f, F = 0.55, *P* = 0.51, baseline vs. cycle 1 vs. cycle 2, 5.24 ± 2.33% vs. 4.66 ± 1.24% vs. 3.98 ± 2.09%). As HLA-DR^+^CD38^+^CD8^+^ T cells were more abundant in the peripheral blood of patients who responded to LEN-TAP conversion therapy, this cell population may serve as an effective biomarker of the therapeutic response to LEN-TAP conversion therapy.

## Discussion

To our knowledge, this is the first and longest follow-up prospective clinical trial to evaluate the efficacy and safety of the triple combination of lenvatinib, TACE and PD-1 inhibitors as conversion therapy for unresectable HCC. In the present study, sequential lenvatinib, TACE and PD-1 inhibitor (LEN-TAP) therapy converted 87.3% of unresectable HCCs to resectable disease, and 59.2% of patients ultimately underwent SLR. Compared with TACE alone as conversion therapy, LEN-TAP conversion therapy significantly improved the SLR rate, ORR, OS, EFS and RFS.

Transarterial chemoembolization (TACE) achieves substantial tumor reduction through localized arterial occlusion and direct cytotoxic effects. Nevertheless, post-procedural hypoxia often stimulates overexpression of pro-angiogenic mediators, including vascular endothelial growth factor (VEGF) and fibroblast growth factor (FGF), which can foster neovascularization and metastatic progression.^7^ Administering lenvatinib, a multitarget kinase inhibitor with activity against VEGF and FGF receptors, offers a potential countermeasure to this adaptive response. Supporting this approach, the LAUNCH trial demonstrated that adding lenvatinib to TACE led to superior clinical benefits in individuals with advanced hepatocellular carcinoma.^9^ In that phase 3 randomized study, median overall survival was 17.8 months (ORR, 54.1%) with lenvatinib plus TACE, compared to 11.5 months (ORR, 25%) with lenvatinib monotherapy. Moreover, salvage liver resection was performed in 15.3% of patients receiving the combined regimen. TACE also enhances tumor immunogenicity through inflammatory activation and antigen release, thereby potentially augmenting the efficacy of immune-based treatments.^11^ Consistent with this, the START-FIT study reported that integrating locoregional and immunotherapeutic strategies produced encouraging results in unresectable HCC^23^ : 55% of participants in that phase 2 trial qualified for curative-intent conversion therapy, and 42% of those achieving radiographic complete response required no further intervention. These observations imply that merging locoregional approaches with tyrosine kinase inhibitors or immunotherapies could expand the subset of patients eligible for sequential curative procedures. Previous clinical studies evaluating lenvatinib plus immunotherapy have also documented promising antitumor responses and a manageable safety profile in the unresectable HCC population,^24^ which could be partially explained by the immunomodulatory activity of lenvatinib and the enhancement of antitumour activity in combination with immunotherapy.^25^ The favorable disease control rate, salvage liver resection rate and long-term outcomes with LEN-TAP treatment in our study further confirmed the physical and chemical effects of TACE in improving tumor localization and, more importantly, increasing the synergistic potential of this triple-therapy strategy.

This study evaluated LEN-TAP conversion therapy in patients with uHCC and well-preserved liver function. No new or unexpected toxicities were observed. The adverse events (AEs) reported were consistent with the established safety profiles of the individual components—lenvatinib, TACE, and PD-1 inhibitors. The most common treatment-related AEs of any grade included abdominal pain, nausea, elevated ALT/AST, decreased appetite, hypertension, and thrombocytopenia. Dose modification or interruption due to AEs was required in 12 patients (16.9%) in the LEN-TAP group. Importantly, no grade 4 or higher AEs occurred, indicating that the toxicity profile of this triple regimen is manageable with appropriate monitoring and dose adjustments.

Over 55% of patients in both cohorts presented with vascular invasion, corresponding to BCLC stage C—the principal form of unresectable HCC. While TACE is not routinely recommended for advanced HCC under the BCLC staging system,^26^ several reports indicate its potential feasibility and efficacy in this setting.^27–29^ Furthermore, Japanese, Korean, and Chinese HCC guidelines endorse TACE as a treatment option for selected patients with branch-type macrovascular invasion.^30–32^ In the current analysis, we enrolled patients with vascular invasion who also had preserved liver function and good performance status. Notably, no cases of hepatic failure or gastrointestinal bleeding were observed in either cohort. Most importantly, in alignment with prior evidence,^9,14,23^ this prospective study demonstrates that the LEN-TAP regimen significantly enhances tumor response and overall survival in patients with advanced HCC.

The perioperative safety profile of patients undergoing salvage liver resection following LEN-TAP conversion therapy was comparable to that of conventional resection, without an increase in overall complications. However, a trend toward prolonged operation time, extended hepatic inflow occlusion, and a relatively higher transfusion rate was noted in the LEN-TAP cohort, potentially attributable to the multimodal nature of the pretreatment regimen. On the other hand, sequential salvage surgery after LEN-TAP conversion therapy plays a vital role in attaining favorable long-term outcomes for uHCC patients. Microvascular invasion (MVI) is a recognized histopathological marker associated with aggressive tumor biology and poorer postoperative prognosis in hepatocellular carcinoma.^33^ This feature is also more frequently observed in cases of advanced HCC. In the current study, the incidence of MVI among advanced HCC patients who underwent salvage resection was 16.7% (7/42), which is lower than rates previously reported by our group and others in early-stage HCC cohorts (ranging from 19.6% to 57.1%).^34,35^ These data imply that LEN-TAP treatment may favorably modulate tumor biology in advanced HCC, thereby lowering the risk of recurrence—a conclusion supported by the prolonged recurrence-free survival (RFS) observed in this trial. Second, salvage resection remains essential for achieving curative intent. Pathological assessment showed that among patients who proceeded to surgery after LEN-TAP, 40.5% (17/42) exhibited a pathological complete response in the resected specimens, while viable tumor cells persisted in the remaining 59.5%, consistent with prior studies.^36,37^ Since residual disease after conversion therapy may lead to relapse, salvage surgery is recommended whenever feasible. Provided resectability criteria are met, pursuing resection likely contributes to the improved event-free and overall survival seen in the LEN-TAP cohort. Thus, for selected uHCC patients, salvage liver resection after LEN-TAP conversion is not only perioperatively safe but also instrumental in enhancing long-term survival.

Through single-cell sequencing and flow cytometry, we demonstrated that patients with a better tumor response had an increased ratio of HLA-DR^+^CD38^+^CD8^+^ T cells in the blood after LEN-TAP conversion therapy. HLA-DR and CD38 molecules are transmembrane glycoproteins that are present on immature T lymphocytes and B lymphocytes. The coexpression of HLA-DR and CD38 on CD8^+^ T cells reflects immune activation.^38^ In our study, HLA-DR^+^CD38^+^CD8^+^ T cells accounted for only a small proportion of the cells in the HC samples; the proportion was much lower than that in the HCC patient samples. Thus, this group of cells may be inactivated under normal conditions. A previous study demonstrated that the coexpression of HLA-DR and CD38 on CD8^+^ T cells is an effective prognostic marker for influenza, HIV infection, and renal cell carcinoma after immunotherapy.^18,39,40^ Consistently, our study demonstrated that the increased proportion of HLA-DR^+^CD38^+^CD8^+^ T cells effectively reflected the response of patients to LEN-TAP therapy. However, the long-term presence of tumor antigens reduces cytokine expression by effector T cells and inhibits reactivation.^41^ Therefore, after the second round of immunotherapy, we did not find significant proliferation of these cells in patients with a PR/CR.

We further observed markedly elevated levels of HLA-DR^+^CD38^+^CD8^+^ T cells infiltration in liver specimens from patients who received conversion therapy followed by salvage resection. Cell–cell communication analysis indicated that this T-cell population mediates antitumor activity through interactions involving the CXCR6 axis and the PI3K–AKT signaling cascade. CXCR6 was initially identified as an HIV co-receptor and is widely present on multiple immune cell types, such as memory T cells, natural killer (NK) cells, NKT cells, dendritic cells, alveolar macrophages, and innate lymphoid cells.^42^ Its specific ligand, CXCL16, occurs in both membrane-bound and secreted isoforms.^42^ Earlier work has highlighted the importance of the CXCL16/CXCR6 pathway in influencing tumor proliferation, migration, and invasiveness.^43,44^ However, CXCR6 expression on T cells in the tumor microenvironment may serve distinct functional roles. Prior research has shown that CXCR6 is a dominant receptor on cytotoxic T lymphocytes (CTLs), irrespective of PD-1 status, which generally differ in their chemokine receptor patterns. The differentiation from stem-like to effector-like CTLs entails a reprogramming of chemokine receptors, notably involving increased CXCR6 expression. Furthermore, the presence of CXCR6, together with IL-15 trans-presentation within the tumor milieu, is critical for sustaining effector-like CTL survival and local proliferation, thereby boosting their antitumor capacity—particularly before terminal exhaustion sets.^45^ In hepatic tissue, CXCL16 produced by sinusoidal endothelial cells attracts and stimulates CXCR6⁺ NKT cells, augmenting antitumor responses through elevated cytokine release such as IFN-γ.^46–49^ In the present study, we detected robust signaling between tumor-derived CXCL16 and CXCR6 on HLA-DR^+^CD38^+^CD8^+^ T cells. Knocking down CXCR6 in this subset considerably impaired T-cell cytotoxicity, indicating that CXCR6 engagement is necessary for effective tumor cell elimination. Notably, CXCR6 suppression also led to diminished expression of key PI3K–AKT pathway elements, along with reduced tumor-killing function. These data imply that CXCR6 likely promotes antitumor responses via activation of the PI3K–AKT cascade. The PI3K pathway is instrumental in CD8⁺ T-cell activation and effector performance, influencing their differentiation, memory maintenance, and antitumor immunity.^50^ Previous reports have correlated low CXCR6 levels with unfavorable outcomes in colorectal cancer and shown that CXCR6 function is closely tied to PI3K/AKT/mTOR pathway activation.^51^ Additional evidence from reproductive biology indicates that exogenous CXCL16 triggers PI3K/AKT signaling in endometrial stromal cells, promoting prolactin secretion—an effect blocked by AKT inhibitors.^52^ These prior observations align with our findings. CXCR6 contributes critically to immune responses mediated by HLA-DR^+^CD38^+^CD8^+^ T cells and may potentiate antitumor immunity by regulating PI3K/AKT signaling. In summary, LEN-TAP treatment appears to amplify immune cell function and signaling through multifaceted mechanisms.

The present study has several limitations. First, in the study design, the treatment for the control cohort was TACE. We preferred lenvatinib over TACE for patients in the control cohort since 40 (56%) of them had BCLC stage C disease; TACE is not recommended for this patient population according to current BCLC guidelines. However, TACE is a recommended option for HCC patients with branched vascular invasion according to several Asian guidelines.^30–32^ The results of several randomized trials initiated in Asia and Europe revealed a survival benefit of TACE in patients with unresectable or even advanced HCC.^53–55^ Owing to the above concerns, TACE was selected for patients in the control cohort in this phase 2 trial. However, lenvatinib should be selected for patients in the control cohort in our future randomized trial. Second, 81.7% of our study cohort had hepatitis B, and whether the results of the present study can be applied to patients with other etiologies is unknown. Third, patients in the LEN-TAP cohort who underwent SLR demonstrated marginally superior RFS than did those in the TACE cohort. This observation will require longer-term follow-up for proper validation. Owing to the unavailability of tumor tissue before LEN-TAP treatment, we ultimately examined changes in immune cells within the PBMC population to indirectly reflect potential immune changes in the liver after LEN-TAP therapy. Although we demonstrated that the increased proportion of HLA-DR^+^CD38^+^CD8^+^ T cells in the blood suggests a response to LEN-TAP therapy, we have not further explored the function of this group of cells.

In conclusion, the results of this trial revealed that the LEN-TAP regimen is safe and effective and may be a promising conversion therapy for patients with unresectable HCC. Moreover, the proportion of HLA-DR^+^CD38^+^CD8^+^ T cells in the blood may be a biomarker for predicting the response to LEN-TAP therapy in these patients.

## Materials and methods

### Study design and participants

This trial was designed to investigate the efficacy and safety of sequential lenvatinib, TACE and PD-1 inhibitors (LEN-TAP) in patients with unresectable HCC; this study was conducted at eight tertiary hospitals in China (details in the supplement). Patients with unresectable HCC were eligible for this trial. The major inclusion criteria were as follows: (1) 18 ~ 70 years of age; (2) HCC diagnosis according to the Chinese Guidelines for the Diagnosis and Treatment of HCC (2019 edition)^30^; (3) BCLC stage B/C; and (4) Child‒Pugh class A/B and Eastern Cooperative Oncology Group (ECOG) performance status (PS) score ≤1. Patients with extrahepatic metastasis, diffuse HCC, or superior mesenteric vein or inferior vena cava tumor thrombi (Vv3) were excluded. A detailed description of the eligibility criteria can be found in the supplement.

This study adhered to the ethical standards outlined in the Declaration of Helsinki and its subsequent revisions. Approval for the study protocol was granted by the Ethics Committee of West China Hospital, Sichuan University (Approval No.: 2020-836). The trial is registered on ClinicalTrials.gov under the identifier NCT04997850. Prior to enrollment, every participant provided written informed consent.

### Interventions

Patients in the TACE monotherapy cohort initiated treatment with TACE one week following enrollment. For those in the LEN-TAP cohort, oral lenvatinib was commenced first, with a starting dose of 12 mg once daily for patients weighing ≥60 kg or 8 mg once daily for those weighing <60 kg. The first TACE session was scheduled two weeks after starting lenvatinib. Intravenous administration of sintilimab or camrelizumab (200 mg)^56,57^ began two weeks post-TACE and was subsequently repeated on a three-week cycle. Treatment with lenvatinib and the PD-1 inhibitor was continued as per protocol until the occurrence of any of the following: Grade 3 or more severe adverse events (AEs), confirmed disease progression, or patient consent withdrawal. While dose reduction was an option for lenvatinib management, it was not permitted for sintilimab or camrelizumab. Temporary treatment interruption for a maximum of 12 weeks was allowed for both agents. Additionally, all patients with hepatitis B virus (HBV) infection received standard antiviral prophylaxis with either entecavir or tenofovir.^30^

All TACE procedures were administered by certified interventional radiologists following a standardized protocol^58^ (see supplement). Briefly, under local anesthesia, catheterization was achieved via the right femoral artery. Initial arteriography of the celiac trunk and superior mesenteric artery was used to map hepatic arterial anatomy. Chemoembolization utilized BSA-adjusted doses of 5-fluorouracil (800–1000 mg) and epirubicin/adriamycin (30–40 mg). Subsequent embolization was achieved via superselective delivery of Lipiodol and polyvinyl alcohol particles into the tumor-supplying segmental hepatic arteries. The volume of embolic agents (5–30 mL) was tailored according to tumor burden, location, and size. Repeat TACE sessions were considered upon confirmation of residual arterial tumor enhancement and adequate liver function reserve.

### Endpoints

The primary endpoint was the rate of successful surgical liver resection (SLR) after conversion therapy (LEN-TAP or TACE). Key secondary endpoints were: objective response rate (ORR by mRECIST, comprising complete or partial responses); overall survival (OS, from treatment initiation to any-cause death); event-free survival (EFS, from treatment start to disease progression, post-SLR recurrence, or death); recurrence-free survival (RFS, from SLR to recurrence or death in resected patients); along with the incidence of adverse events post-conversion and perioperative complications after SLR, all of which were prospectively documented.

### Efficacy assessment and adverse events

Tumor response and resectability were assessed at 8 ± 2-week intervals during conversion therapy (LEN-TAP or TACE). Each evaluation included contrast-enhanced computed tomography (CT), with responses graded according to mRECIST and RECIST v1.1. For safety monitoring, serial laboratory tests were performed, including complete blood counts and assessments of hepatic, renal, thyroid, and myocardial function. All follow-up assessments were conducted by experienced surgeons at the respective participating centers. Adverse events (AEs) were systematically documented and graded based on the National Cancer Institute Common Terminology Criteria for Adverse Events (CTCAE v5.0). Treatment decisions, including initiating second-line therapy for non-responding patients or scheduling surgery for those meeting conversion criteria, were determined through weekly investigator meetings and multidisciplinary team (MDT) discussions.

### The criteria for resectability

Hepatic tumor resectability was determined based on the following criteria: (1) tumor response: intrahepatic target lesions must demonstrate a sustained response (complete response, partial response) or stable disease for a minimum duration of 2 months; (2) future liver remnant (FLR): the ratio of future liver remnant volume to standard liver volume (FLR/SLV) must exceed 40% for patients with underlying cirrhosis, or 30% for those with normal liver parenchyma; (3) hepatic functional reserve: patients were required to have well-preserved liver function, indicated by a Child-Pugh class A and an indocyanine green retention rate at 15 minutes (ICG-R15) of less than 20%; (4) performance status: An Eastern Cooperative Oncology Group (ECOG) Performance Status score of 0 or 1 was required.

### Salvage liver resection, adjuvant therapy and follow-up

Patients who achieved successful conversion from unresectable to resectable hepatocellular carcinoma (HCC) became eligible for salvage liver resection (SLR). A mandatory preoperative washout period was observed: PD-1 inhibitor therapy was withheld for a minimum of 3 weeks, and lenvatinib was discontinued for 1 week prior to surgery. The magnitude of resection was classified as major (involving ≥3 Couinaud’s segments) or minor ( < 3 segments). Postoperative morbidity was assessed using the Clavien-Dindo classification. Pathologic outcomes were evaluated, with a pathologic complete response (pCR) indicating no residual viable tumor, and a major pathologic response (MPR) denoting viable tumor cells occupying ≤10% of the total tumor bed area.^59^

Per MDT recommendations, adjuvant therapy with the original conversion regimen or its key components was advised for over six months post-SLR, with dose adjustments permitted based on the patient’s clinical status, treatment tolerance, and adverse events. Therapy discontinuation could be considered only after two consecutive imaging assessments (spaced at least 3 months apart) confirmed no recurrence or metastasis, coupled with normalized tumor marker levels sustained over the same period. Postoperative surveillance consisted of quarterly follow-up visits. Each visit included a physical examination, laboratory tests (complete blood count with differential, AFP, DCP, and liver function panels), and contrast-enhanced CT imaging of the chest and abdomen.

### Biomarker analysis

#### Single-cell RNA sequencing (scRNA-seq)

We applied scRNA-seq to profile both peripheral blood mononuclear cells (PBMCs) and dissociated liver tissue cells. Library preparation was conducted with the 10x Genomics Chromium Single Cell 3’ Kit v3, aiming to capture around 5000 individual cells per sample. PBMC isolation was achieved via standard density gradient centrifugation. For liver tissues, samples were first finely minced into 1–2 mm³ pieces. A enzymatic dissociation step was then performed by incubating the fragments at 37 °C for 30–60 min in a solution containing collagenase (0.1–1 mg/mL) and DNase I (20–50 U/mL). The dissociated cell mixture was subsequently passed through a 40 µm cell strainer to generate a single-cell suspension. Prior to loading onto the 10x Chromium controller, cell viability was confirmed for all samples. Subsequent steps, including cDNA synthesis and library preparation, strictly followed the manufacturer’s protocol. The final libraries were sequenced on an Illumina NovaSeq 6000 platform (LC-Bio, Hangzhou, China) to generate 150 bp paired-end reads, ensuring a minimum depth of 20,000 reads per cell. Initial data processing involved demultiplexing raw sequencing data and format conversion to FASTQ using bcl2fastq (v2.20). Further processing, encompassing barcode assignment, UMI counting, and alignment to the Ensembl GRCh38 reference genome, was executed with Cell Ranger (v3.1.0).

For the liver tissue dataset, we implemented a quality control filter to exclude cells expressing fewer than 200 or more than 10,000 genes, or those with mitochondrial gene content surpassing 20%. All downstream analyses utilized the Seurat package (v3.1.1 for PBMC data). The analytical workflow included data log-normalization, principal component analysis (PCA; utilizing the top 10 principal components), and nonlinear dimensionality reduction (t-SNE applied to PBMCs; UMAP applied to liver tissue). Cell clusters were identified via a graph-based clustering algorithm relying on shared nearest neighbors (SNN).

Specifically for PBMC data, T cells were subjected to reintegration and reclustering using parameters set to k.filter=20 and Resolution=3.2. A further sub-clustering analysis was conducted on the HLA-DR⁺CD38⁺ T cell subset using k.filter = 20 and resolution = 0.8. Cell type annotation for liver samples was performed by integrating the Scibet tool with well-established marker genes. To identify cluster-specific marker genes in the PBMC dataset, we employed the Wilcoxon rank-sum test, applying a threshold requiring genes to be detected in over 10% of cells within a cluster and to exhibit an average log₂ fold change greater than 0.26. Differential expression analysis between conditions was carried out using a bimodal test. The significance thresholds were set as follows: for PBMCs, an adjusted p-value < 0.01 and an absolute log₂ fold change ( | log₂FC | ) ≥ 0.26; for liver cells, |log₂FC | ≥ 0.25 and an adjusted p-value ≤ 0.05.

Functional enrichment analysis of the resulting differentially expressed genes (DEGs) for Gene Ontology (GO) terms and Kyoto Encyclopedia of Genes and Genomes (KEGG) pathways was performed with the R package ClusterProfiler. In PBMC samples, this analysis specifically targeted DEGs in HLA-DR⁺CD38⁺CD8⁺ T cells when comparing pre- and post-treatment states. For the liver dataset, intercellular communication networks and ligand-receptor interactions among different cell populations were inferred using the CellChat tool.

#### Flow cytometry analysis

Peripheral blood was drawn from patients at three distinct phases: before the commencement of treatment, after the first dose of PD-1 inhibitors, and following the second administration. The subsequent flow cytometric analysis adhered strictly to the protocols provided by the reagent manufacturers. Lymphocytes were isolated from the collected blood samples and subjected to staining with a predefined antibody cocktail, the composition of which is provided in Supplementary Table S1. In cases where intracellular markers were analyzed, cell fixation and permeabilization were carried out employing the FOXP3 Transcription Factor Staining Buffer Set from eBioscience. A BD flow cytometer equipped with FACSDiva software was used for data collection, and the resulting data were processed and analyzed using FlowJo software.

#### H&E Staining and Immunofluorescence Analysis

Liver tumor samples from two HCC patient cohorts (n = 3 per cohort: LEN-TAP conversion or direct resection) were fixed in 4% PFA, processed through dehydration and paraffin embedding, and sectioned. H&E staining involved hematoxylin staining (5–10 min), differentiation, bluing, eosin counterstaining (30 s-2 min), dehydration, and mounting for light microscopy.

For IF, sections were dewaxed and subjected to antigen retrieval in EDTA buffer (pH 8.0) at 95 °C for 15–20 min. After blocking with 10% goat serum (1 h, RT), sections were incubated overnight at 4 °C with fluorophore-conjugated primary antibodies against GZMB (Abcam), HLA-DR (Abcam), CD38 (Starter), and CD8 (ZSGB-BIO). Nuclei were counterstained with DAPI, slides were mounted with anti-fade medium, and imaging was performed on a fluorescence microscope.

#### In vitro T-cell culture and expansion

T cells were expanded in vitro by culture in T-cell expansion serum-free medium (SFM, Thermo Fisher Scientific, USA) within 24-well plates. Cell activation was achieved with Dynabeads™ Human T-Activator CD3/CD28 (Thermo Fisher Scientific, USA) at a 1:1 bead-to-cell ratio, and the culture was supplemented with IL-2 (30 ng/mL) and IL-15 (10 ng/mL; Thermo Fisher Scientific, USA) to facilitate proliferation. Incubation was carried out under standard conditions (37 °C, 5% CO₂) in a humidified atmosphere.

#### siRNA transfection

A CXCR6-targeting siRNA (siCXCR6) and a control siRNA (NC-siRNA) were designed against the human CXCR6 sequence (RefSeq: NM_006564.4). The siCXCR6 duplex (Fuji Bio, China) sequences were: Sense, 5′-GUUUCAUUGUAGUGGUUAA-3′; Antisense, 5′-UUAACCACUACAAUGAAAC-3′. CD8⁺HLA-DR⁺CD38⁺ T cells in Opti-MEM were transfected with 50 nM siRNA via Lipofectamine RNAiMAX (Thermo Fisher Scientific, USA). Subsequent RNA extraction utilized TRIzol reagent (Invitrogen, USA), with concentration determined by Qubit. From 1 μg of RNA, cDNA was synthesized by reverse transcription (42 °C for 30 min; 70 °C for 5 min). qRT-PCR was conducted in 20 μL reactions containing 10 μL of 2× SYBR Green Master Mix, 0.4 μL of each primer (10 μM), 1 μL of cDNA, and nuclease-free water. The thermal profile included: 95°C for 5 min; 40 cycles of 95 °C/10 s, 60 °C/30 s, 72 °C/30 s, with fluorescence acquisition at each cycle’s end. Melting curve analysis confirmed specificity. CXCR6 expression was normalized to GAPDH and quantified by the ΔΔCt method.

#### Coculture model of T cells and tumor cells in vitro

For the in vitro T cell-tumor cell coculture, 96-well plates were pre-coated overnight at 4 °C with a solution of anti-CD3 and anti-CD28 antibodies (each 5 μg/mL in PBS; Thermo Fisher Scientific, USA). After two washes with serum-free medium to clear excess antibodies, activated HLA-DR⁺CD38⁺CD8⁺ T cells and Huh7 cells (Shanghai Institute of Life Sciences, China) were plated together at a 5:1 ratio. The culture was carried out in medium containing IL-2 (30 ng/mL) and IL-15 (10 ng/mL; Thermo Fisher Scientific, USA) and was sustained for 48–72 hours under standard conditions (37 °C, 5% CO₂), aligning with the monoculture setup.

#### Western blotting

Post-coculture, cells were PBS-washed and lysed in RIPA buffer with 1% PMSF (Biyuntian Biotechnology, China). Protein concentrations were quantified via a BCA assay. Lysates were boiled, separated by SDS-PAGE (90 V, 30 min; then 120 V, 90 min), and transferred to PVDF membranes (90 V, 1 h). The membranes were blocked with 3–5% BSA/TBST (90 min, 37 °C) before overnight incubation at 4 °C with primary antibodies (diluted in 3–5% BSA/TBST; Abcam, UK) targeting GAPDH, BAD, PI3K, p-PI3K, AKT, and p-AKT. After washing, incubation with HRP-conjugated secondary antibodies was performed, followed by another wash and chemiluminescent development.

### Statistical analysis

Statistical analyses were performed using SPSS (version 25.0; IBM Corp.) and R (version 4.0.2). The study’s sample size was calculated a priori according to the primary endpoint via a log-rank test (detailed in the Supplement). For data presentation, continuous variables are summarized as medians with interquartile ranges (IQRs) and were compared using either Student’s t-test or the Mann–Whitney U test, depending on their distribution. Categorical variables are described as numbers and percentages, with group comparisons made by the chi-square test or Fisher’s exact test. Survival outcomes, including overall survival (OS) and event-free survival (EFS), were visualized with Kaplan-Meier curves, and the log-rank test was applied to evaluate inter-group differences. Across all tests, statistical significance was defined as a two-sided p-value of less than 0.05.

## Supplementary information

Supplementary information

Supplementary information

SIGTRANS-15441-s03

SIGTRANS-15441-s04

SIGTRANS-15441-s05

SIGTRANS-15441-s06

## Data Availability

The datasets underpinning the conclusions of this research are included in this published article and its supplementary materials. All raw sequencing data generated in this study have been submitted to the Genome Sequence Archive (GSA) at the National Genomics Data Center, China National Center for Bioinformation / Beijing Institute of Genomics, Chinese Academy of Sciences. The accession number for the dataset is HRA013410, and it is available to the public via the following URL: https://ngdc.cncb.ac.cn/gsa.
