# Supplementary material for: Lenvatinib plus transarterial chemoembolization and PD-1 inhibitors as conversion therapies for unresectable intermediate-advanced hepatocellular carcinoma: a phase 2 trial and exploratory biomolecular study
Source: Signal Transduct Target Ther. 2026 Jan 22;11:37. doi: 10.1038/s41392-025-02498-z (PMC12823698; doi:10.1038/s41392-025-02498-z)
Supplement: Supplementary file 1 — Supplementary information [file 41392_2025_2498_MOESM1_ESM.docx]

Supplementary Materials for

Lenvatinib Plus Transarterial Chemoembolization And PD-1 Inhibitors As Conversion Therapies For Unresectable Intermediate-advanced Hepatocellular Carcinoma: A Phase 2 trial And Exploratory Biomolecular Study

Xiaoyun Zhang^1,^^†^, Haozheng Cai^1,†^, Wei Peng^1,2,†^, Haiqing Wang^3,†^, JiaYi Wu^4^, Xinrui Zhu^1,5^, Weixin Guo^6^, Fei Xie^7^, Yu Zhang^8^, Ming Wang^9^, Yu Yu^10^, Yongjie Zhou^11^, Chuan Li^1^, Junyi Shen^1^, Chang Liu^1^, Yu Yang^12^, Xiaozhong Jiang^10^, Qiu Li^12^, Weixia Chen^13^, Yujun Shi^14^, Wusheng Lu^1^, Xin Sun^2^, Xielin Feng^3^, Maolin Yan^4^, Shuqun Cheng^6^ , Tianfu Wen^1 *^and The Chinese Association of Liver Cancer (CALC)

Correspondence to: wentianfu@scu.edu.cn

**This PDF file includes:**

Materials and Methods

Figures. S1 to S4

Tables S1 to S5

References

**Materials and methods**

**Rigorous review process details**

West China hospital’s rigorous review process approved this study. Firstly, this novel conversion strategy for unresectable HCC was approved as a surgical innovation by medical administration department of West China Hospital in April 2020. Off-label use of Sintilimab/Camrelizumab in LEN-TAP strategy for newly diagnosed unresectable HCC was approved by pharmaceutical management committee of West China Hospital in Aug 2020 after a second review since they were approved for previously treated advanced HCC by NMPA. The study protocol conformed to the ethical guidelines of the 1975 Declaration of Helsinki was subsequently approved by ethics committee of West China Hospital (Approval Number: 2020-836). This trial was registered with ClinicalTrial.gov (NCT04997850). Informed consent was obtained from all patients.

**List of medical centers participated in this trial**

West China Hospital (Chengdu, China), Fujian Provincial Hospital (Fuzhou, China), Sichuan Provincial People's Hospital (Chengdu, China), Sichuan Cancer Hospital & Institute (Chengdu, China), First People's Hospital of Neijiang (Neijiang, China), Eastern Hepatobiliary Surgery Hospital (Shanghai, China), The Third People's Hospital of Chengdu (Chengdu, China), Second People’ s Hospital of Yibin (Yibin, China).

**Sample size**

We estimated the sample size of 142 patients which was based on the assumption that 30% of patients who received LEN-TAP conversion therapy would be eligible for liver resection, while 10% of patients who received TACE mono would be eligible for liver resection (α=0.05; β=0.8; 10% for successful conversion resection rate in TACE group; 30% for successful conversion resection rate in triple combination group; total sample size of 118 patients, with an additional 24 patients to allow for dropout or other reasons). Sample size calculation was performed by PASS (version 15, NCSS, LLC. Kaysville, Utah, USA).

**Inclusion and exclusion criteria**

**Inclusion Criteria were as follows**:

(1) 18 years old ≤ age ≤ 70 years old, no gender limit;

(2) HCC patients who are in strict compliance with the clinical diagnostic criteria of the "Guidelines for the Diagnosis and Treatment of Hepatocellular Carcinoma (2019 Edition) or confirmed by histopathology or cytology;

(3) Have not received any systemic treatment for HCC in the past.

(4) ECOG PS score 0-1;

(5) Child-Pugh liver function rating A or B;

(6) Patients with BCLC stage B and C liver cancer;

(7) According to the evaluation criteria for the efficacy of solid tumors (mRECIST), there is at least one imaging measurable lesion;

(8) If the patient is HBsAg positive, HBV-DNA will be less than 2000 IU/ml (10000 copies/ml) during PD-1antibody treatment;

(9) The function of major organs is normal, that is, it meets the following standards: i) sufficient bone marrow function, defined as absolute neutrophil count (ANC) greater than or equal to 1.5×10^9 per liter (/L); ii) hemoglobin (Hb) greater than or equal to 8.5 grams per deciliter (g/dL); iii) platelet count greater than or equal to 75×10^9/L); iv) Sufficient liver function, defined as: aspartate aminotransferase (AST), alkaline phosphatase (ALP) and alanine aminotransferase (ALT) are less than or equal to 5 ULN; v) Sufficient coagulation function, defined as the International Normalized Ratio (INR) less than or equal to 2.3; vi) Sufficient renal function is defined as a creatinine clearance rate greater than 40 milliliters per minute (mL/min), calculated according to Cockcroft and Gault formulas; vii) Sufficient pancreatic function, defined as amylase and lipase≤1.5×ULN; viii) Normal thyroid function is defined as thyroid stimulating hormone (TSH) within the normal range, If the baseline TSH is outside the normal range, subjects whose total T3 (or FT3) and FT4 are within the normal range can also be included in the group.

(10) Use up to 3 antihypertensive drugs to adequately control blood pressure (BP), defined as BP lower than 150/90 mmHg at the time of screening, and there is no change in antihypertensive treatment within 1 week before cycle 1/day 1.

(11) The patient is expected to survive more than 3 months.

(12) No pregnancy or pregnancy plans.

(13) The subjects voluntarily joined the study and signed an informed consent form, with good compliance and cooperation with follow-up.

**Exclusion Criteria:**

(1) Extrahepatic metastasis of primary liver cancer;

(2) Diffuse liver cancer, intrahepatic tumor burden ≥50%; portal vein tumor thrombus (superior mesenteric vein tumor thrombus, type IV), inferior vena cava tumor thrombus;

(3) Contraindications of TACE and epirubicin;

(4) Those who have participated in other clinical trial drugs within 4 weeks;

(5) Those who are known to be allergic to the ingredients of lenvatinib;

(6) Those who are known to be allergic to the active ingredients or excipients of sintilizumab and camrelizumab;

(7) A history of liver resection, liver transplantation, interventional therapy, and other malignant tumors;

(8) Women who are pregnant or breast-feeding; those with fertility who are unwilling or unable to take effective contraceptive measures;

(9) Patients with grade Ⅱ or higher myocardial ischemia or myocardial infarction, poorly controlled arrhythmia (including QTc interval ≥470 ms); according to NYHA standards, grade Ⅲ to Ⅳ cardiac insufficiency, or cardiac color Doppler ultrasound examination suggests left ventricular ejaculation Blood score LVEF<50%;

(10) Abnormal coagulation function (INR>1.5 or prothrombin time (PT)>ULN+4 seconds or APTT>1.5 ULN), have bleeding tendency or are receiving thrombolytic or anticoagulant therapy;

(11) Have a mental illness or a history of psychotropic drug abuse;

(12) Combined with HIV-infected patients;

(13) Known allogeneic organ transplantation (except corneal transplantation) or allogeneic hematopoietic stem cell transplantation;

(14) Patients with active infection;

(15) Patients with poor compliance such as floating population;

(16) Have received the following therapies in the past: anti-PD-1, anti-PD-L1 or anti-PD-L2 drugs or for another stimulating or synergistic inhibition of T cell receptors (for example, CTLA-4, OX-40, CD137) medicine;

(17) An active autoimmune disease that requires systemic treatment (such as the use of disease-relieving drugs, glucocorticoids, or immunosuppressive agents) occurred within 2 years before the first administration. Replacement therapies (such as thyroxine, insulin, or physiological glucocorticoids for adrenal or pituitary insufficiency, etc.) are not considered systemic treatments;

(18) Are receiving systemic glucocorticoid therapy (excluding nasal spray, inhalation or other local glucocorticoids) or any other form of immunosuppressive therapy within 7 days before the first administration of the study; Note: physiological doses are allowed Glucocorticoids (≤10 mg/day prednisone or equivalent);

(19) There is clinically uncontrollable pleural effusion/abdominal effusion (patients who do not need to drain the effusion or stop drainage for 3 days without a significant increase in effusion can be included in the group);

(20) Acute or chronic active hepatitis B or C infection, HBV DNA ≥ 200000IU/ml or 106 copies/ml when Sintilimab is treated; hepatitis C virus HCV RNA ≥ 103 copies/ml; Hepatitis B surface anti-(HBsAg) and anti-HCV antibodies are positive at the same time.

(21) Live vaccine has been vaccinated within 30 days before the first dose (cycle 1, day 1); Note: It is allowed to receive the inactivated virus vaccine for seasonal influenza injection within 30 days before the first dose; but it is not allowed to receive intranasal vaccine Medicated live attenuated influenza vaccine.

(22) The researcher believes that it is not suitable for inclusion in the group.

**TACE procedure**

TACE was performed through the right femoral artery with local anesthesia. After arteriography of the celiac trunk and superior mesenteric artery to visualize arterial vascularization of the liver, body surface-dependent doses of the chemotherapeutic agents 5-fuorouracil (800~1000 mg) and epirubicin-adriamycin (30~40 mg) were injected. Subsequently, lipiodol and polyvinyl alcohol foam embolization particles were injected as selectively as possible into the hepatic segmental artery at the target tumor location. The embolization agent doses ranged from 5 to 30mL and were determined based on the tumor location, size, and number. On demand TACE may be repeated based on the evidence of remnant hepatic arterial blood supply and liver function.

**Efficacy assessment and adverse events**

Patients were followed up every 8±1 week during LEN-TAP or TACE conversion therapy. Tumor response and resectability assessment were performed via enhanced CT using the response evaluation criteria in solid tumors, version 1.1 (RECIST 1.1) and the modified response evaluation criteria in solid tumors (mRECIST) every 8±2 weeks^1,2^. Several measures were raised up to protect patient safety. Blood cell counts, parameters of hepatic, renal, thyroid and myocardial enzymes were regularly monitored. Follow-up during conversion therapy was performed by an experienced surgeon from each center. Each adverse event (AE) was assessed and graded using the National Cancer Institute Common Terminology Criteria for Adverse Events version 5.0. Weekly conference within investigators and multidisciplinary team (MDT) consultation was held to determine the second-line therapy for patients who failed to achieve conversion and to determine the surgery schedule for patients who achieved successful conversion.

**Quantification of Cytokines**

Blood was collected from uHCC patients, and serum were freshly isolated by centrifuge at 3,500 rpm for 10 minutes and then preserved in liquid nitrogen. For accurate detection of cytokines in patient serum, R&D (LKTM014B) Human XL Cytokine Luminex® Performance Assay 46-plex Fixed Panel was applied following the instructions provided by the manufacturer.

**Data and Statistical Analysis**

Continuous data are presented as the mean ± standard deviation. Comparison of cell proportions before and after treatment in the same patient using paired t-tests. Patients with different prognosis were compared by unpaired T test. Comparison of samples between multiple groups using one-way analysis of variance. GraphPad Prism 9.0 was used for statistical analysis. Statistical significance was set at P < 0.05.

**Comparison of Camrelizumab and Sintilimab as PD-1 Inhibitors**

Camrelizumab and Sintilimab, as PD-1 inhibitors, demonstrated no significant differences across multiple aspects. In terms of baseline characteristics, including age, gender, etiology, ECOG PS, Child-Pugh score, tumor number, largest tumor size, vascular invasion, and AFP levels, the two groups showed comparable results (Table S2). Regarding antitumor activity, both inhibitors achieved similar rates of objective response, disease control, and amenability for salvage liver resection under RECIST 1.1 and mRECIST criteria (Table S3). Additionally, the incidence of adverse events, both overall and grade 3, was comparable between the two groups (Table S4). Finally, perioperative outcomes for patients undergoing salvage liver resection, including hospital stays, operation duration, intraoperative blood loss, transfusion rates, pathological response, and Clavien-Dindo classification, also showed no statistically significant differences (Table S5). These findings suggest that Camrelizumab and Sintilimab are similarly effective and safe.

A total of 15 (39.5%) patients in the Camrelizumab group and 16 (51.5%) patients in the Sintilimab group died, and both the OS, RFS and EFS were comparable between two groups (P=0.28, P=0.95 and P=0.35, Figure S1).

**Cytokine alterations after LEN-TAP therapy**

Additionally, 46 cytokines were successfully detected and analysed in patients in the LEN-TAP cohort. Although no discernible/obvious differences were found between the CR/PR and SD/PD groups either before or after LEN-TAP conversion therapy, several cytokines showed great specific differences within the CR/PR or SD/PD group. Specifically, the Flt-3 ligands MIP-3α and IL-6 were obviously differentially expressed only in the CR/PR group (68.15 ± 29.53 vs. 109.5 ± 58.83; 94.80 ± 130.3 vs. 26.55 ± 20.98; 9.401 ± 10.90 vs. 58.04 ± 117.0; CR/PR before LEN-TAP treatment (n=24) vs. CR/PR after LEN-TAP conversion therapy (n=34), respectively; Supplementary Figure S4A), whereas TNF-β, IL-17A and TRAIL were highly expressed in the SD/PD group samples after LEN-TAP conversion therapy (n=8) compared with the SD/PD group samples before LEN-TAP conversion therapy (n=21), as shown in Supplementary Figure S4B. The relationship between the levels of these cytokines and the clinical response after LEN-TAP conversion therapy remains elusive, but this panel, which includes Flt-3 ligand, MIP-3α, IL-6, TNF-β, IL-17A and TRAIL, may be a potential indicator of patient response.

In our study, we observed distinct patterns of cytokine level changes in patients undergoing LEN-TAP treatment. Specifically, the levels of FLT-3 ligand (FLT3L) and interleukin-6 (IL-6) were significantly increased in the blood of patients who achieved CR/PR, whereas the levels of tumour necrosis factor-beta (TNF-β), interleukin-17A (IL-17A), and TNF-related apoptosis-inducing ligand (TRAIL) were markedly elevated in patients with SD/PD. Additionally, the levels of macrophage inflammatory protein-3 alpha (MIP-3α, also known as CCL20) significantly decreased in the CR/PR group following treatment.

FLT3L is a critical haematopoietic growth factor required for the development of dendritic cells (DCs), including both plasmacytoid DCs (pDCs) and type-1 conventional DCs (cDC1s) ^3,4^. Its therapeutic potential has been highlighted in clinical trials in which FLT3L was used to mobilize DCs and enhance antitumour immunity in patients. The elevated levels of FLT3L in responders suggest that LEN-TAP may facilitate the expansion and activation of DCs populations, thereby promoting effective immune responses against HCC. IL-6 plays a dual role in immunity. It promotes the differentiation of Th17 cells in the presence of TGF-β while simultaneously inhibiting TGF-β-induced Tregs^5^. The increased IL-6 levels in the CR/PR group may reflect a form of "immune reprogramming," indicating enhanced proinflammatory and anti-tumour immunity following treatment. In contrast, TNF-β, a proinflammatory cytokine involved in immune regulation^6^, was elevated in nonresponders (those with SD/PD), possibly reflecting a state of chronic inflammation or immune tolerance that impedes effective tumour clearance. Similarly, IL-17A—secreted by Th17 cells—has been shown to promote tumour progression by enhancing angiogenesis, proliferation, metastasis, and resistance to apoptosis^7^. Elevated IL-17A levels in SD/PD patients are consistent with the formation of an immunosuppressive, tumour-promoting microenvironment and are negatively correlated with the prognosis of patients with HCC^8^. TRAIL is known for its selective cytotoxicity against tumour cells without harming normal tissues^9^. However, intrinsic resistance to TRAIL-induced apoptosis has been reported in HCC cells, which may explain the paradoxical elevation of TRAIL in nonresponders as a failed attempt at tumour cell elimination. Interestingly, MIP-3α, a chemokine that recruits immature DCs and lymphocytes and has been associated with inflammation and tumorigenesis^10^, was significantly reduced in CR/PR patients. Its downregulation may indicate a shift in the immune microenvironment from inflammatory cell recruitment t towards an antitumour immune state, with diminished mobilization of tumour-promoting immune subsets.

**Figure. S1.**
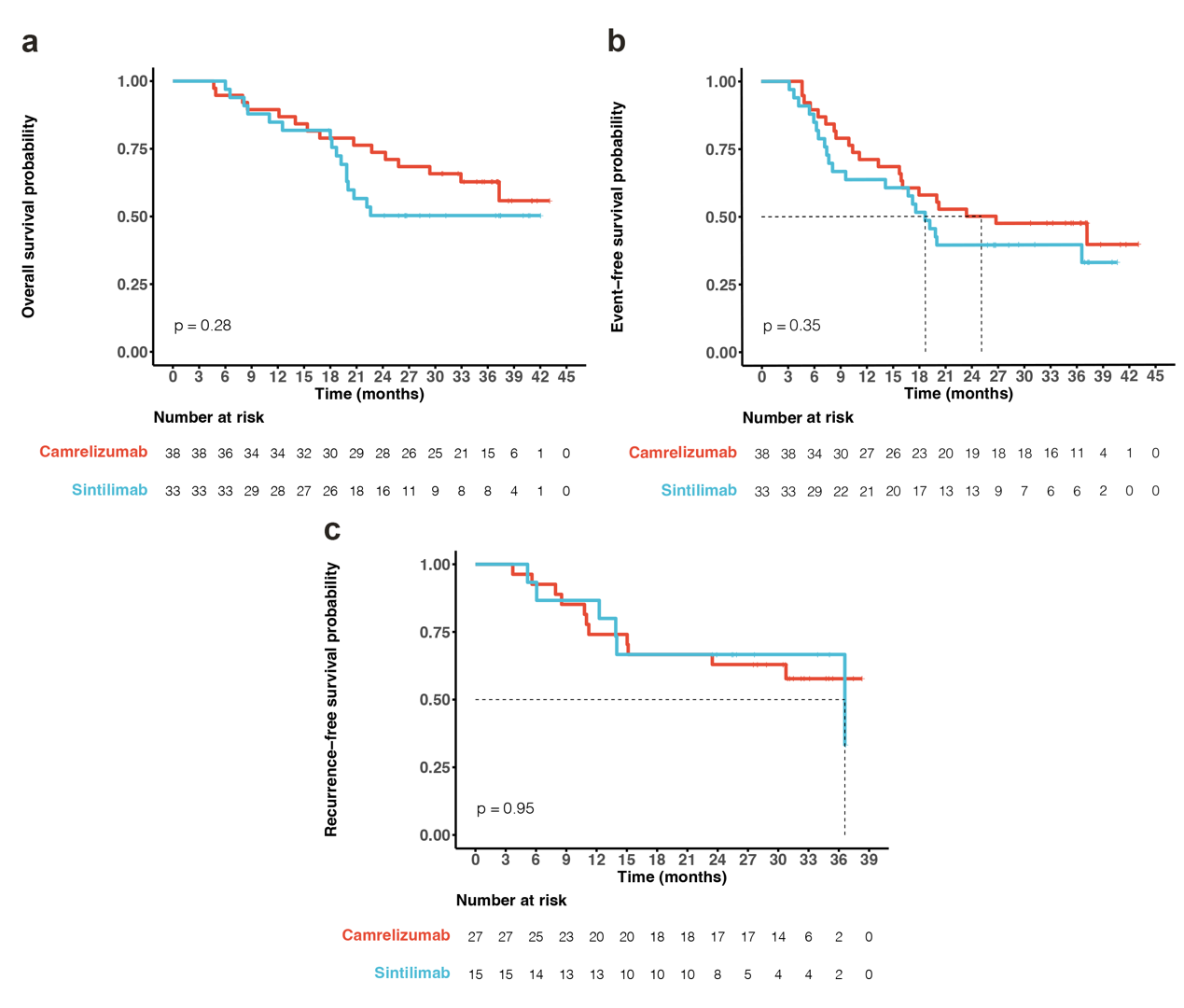
Figure S1. **Kaplan–Meier curves of OS (a), EFS (b) and RFS (c) between the Camrelizumab and Sintilimab groups.** OS, overall survival; EFS, event-free survival; RFS, recurrence-free survival.

**Figure. S2.**


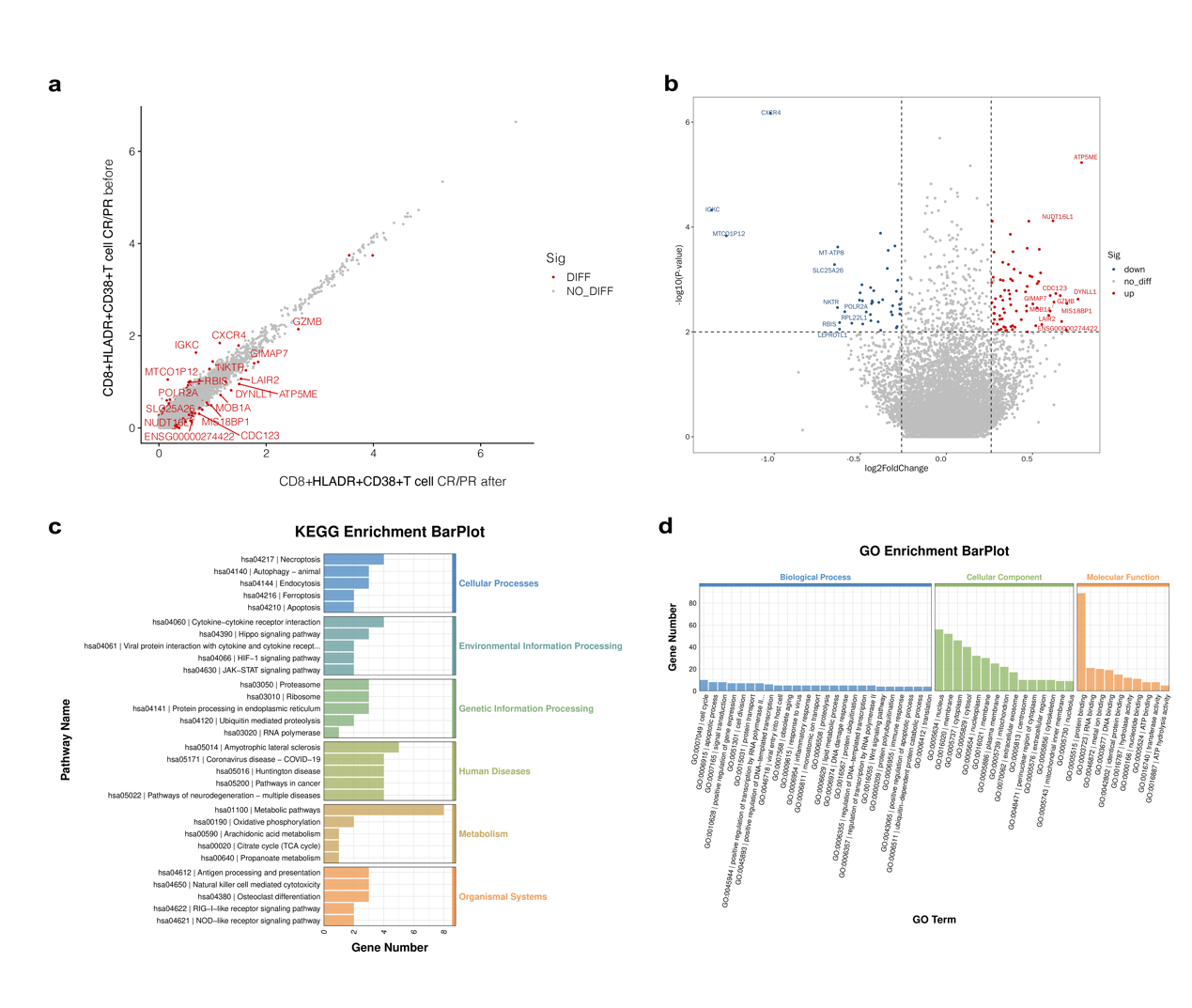


**Figure S2. The differentially expressed genes (DEGs) and the related enrichment analysis in HLA-DR⁺CD38⁺CD8⁺ cells. a** MA plot of differentially expressed genes in HLA-DR⁺CD38⁺CD8⁺ T cells from PR/CR patients before and after treatment; **b** Volcano plot of differentially expressed genes in HLA-DR⁺CD38⁺CD8⁺ T cells from PR/CR patients before and after treatment; **c** Bar chart of KEGG pathway enrichment analysis for differentially expressed genes in HLA-DR⁺CD38⁺CD8⁺ T cells from PR/CR patients before and after treatment; **d** Bar chart of GO functional enrichment analysis for differentially expressed genes in HLA-DR⁺CD38⁺CD8⁺ T cells from PR/CR patients before and after treatment.

**Figure. S3.**


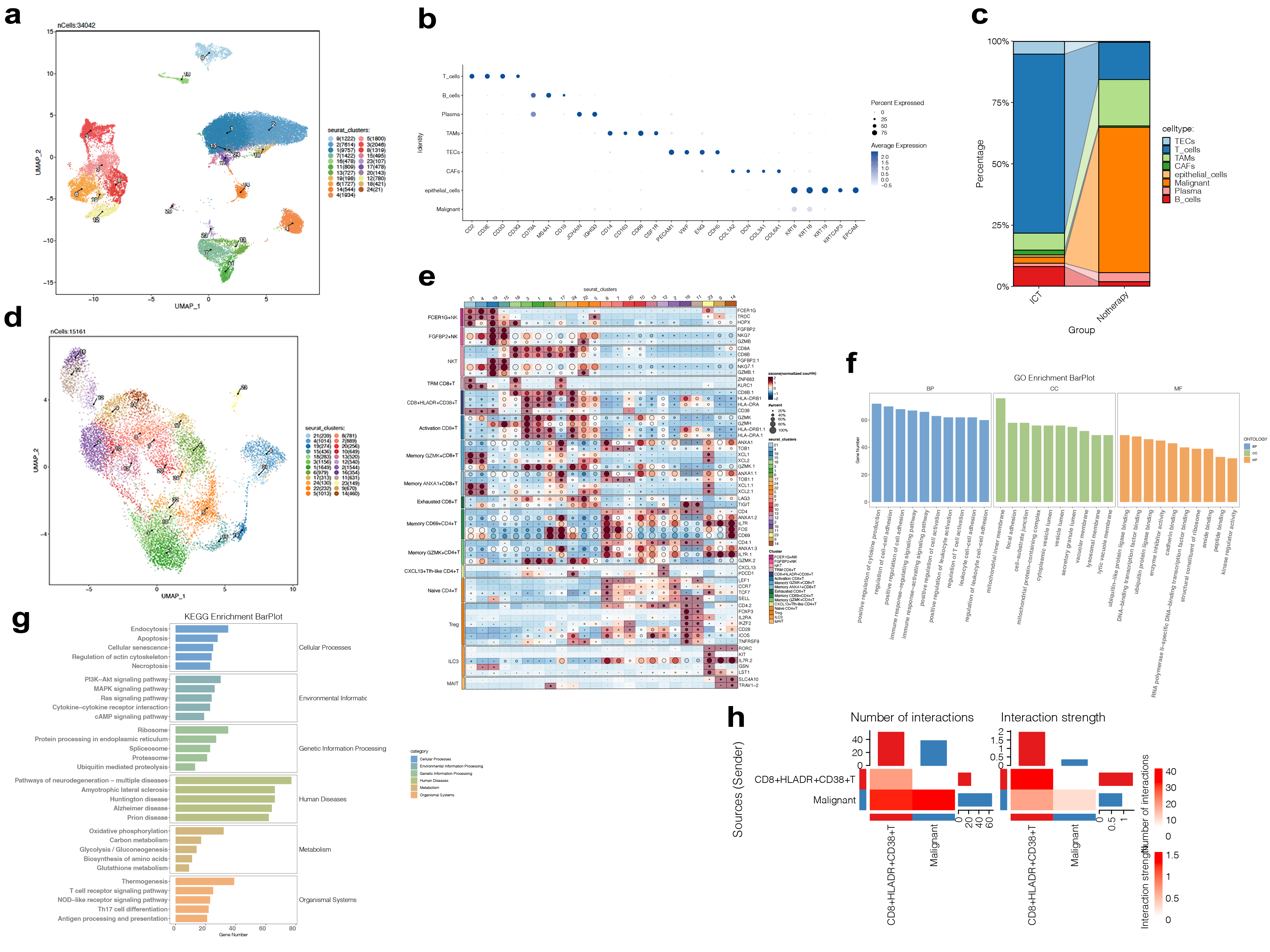


**Figure S3. Single-cell RNA sequencing analysis of uHCC tumor microenvironment. a** UMAP dimensionality reduction analysis shows the distribution of different cell subpopulations. **b** Heatmap of cell clustering marker genes.**c** UMAP dimensionality reduction analysis shows the distribution of different T cell subpopulations. **d** T cell subset clustering marker gene heatmap. **e** Differential gene GO enrichment bar chart. **f** Differential gene KEGG pathway enrichment bar chart. **g** Heatmap of communication differences between HLA-DR⁺CD38⁺CD8⁺ T cells and tumor cell clusters.

**Figure. S4.**


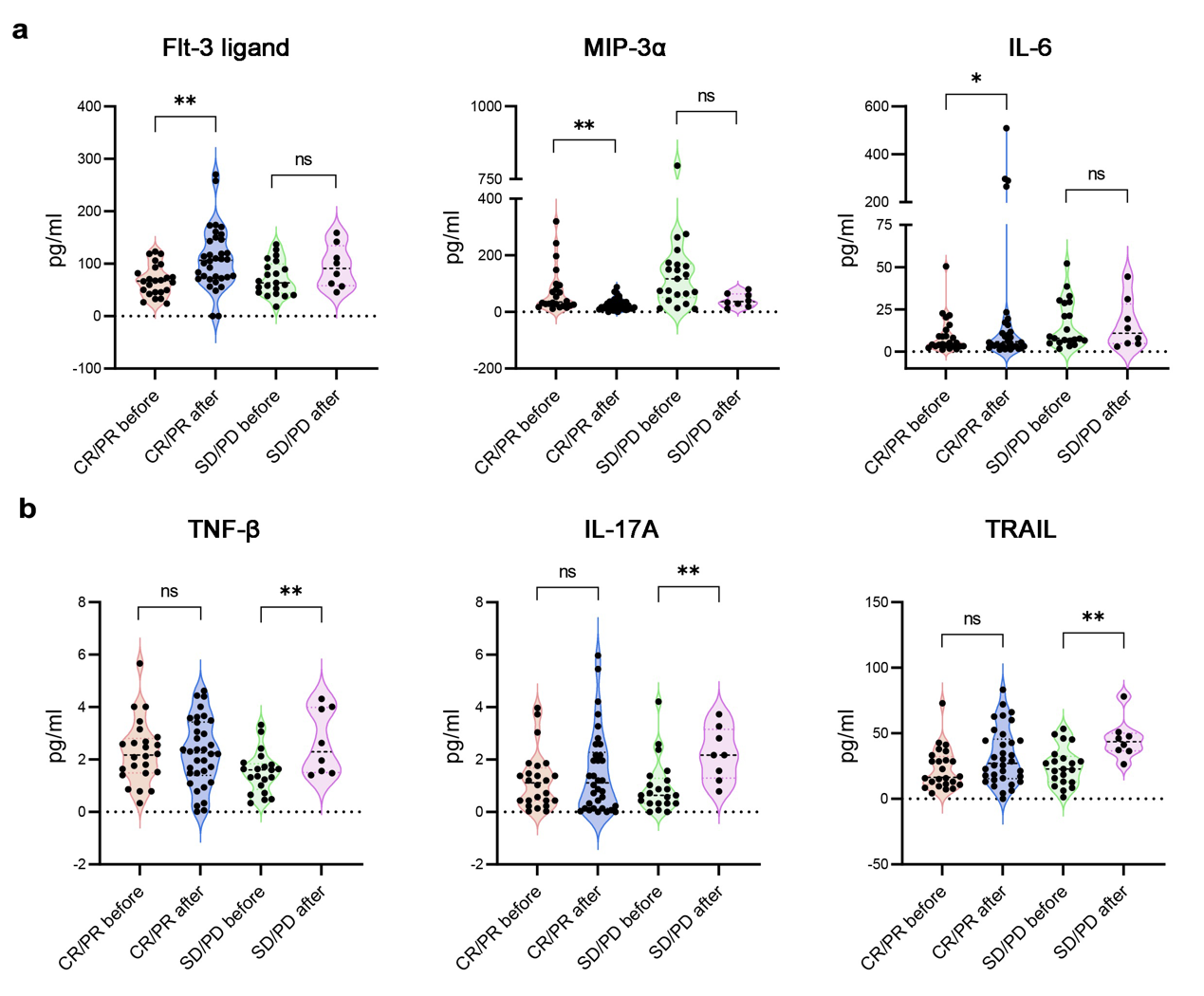


**Figure S4. The level of cytokines in patients before and after LEN-TAP treatment. a** Cytokines significantly changed in CR/PR groups. **b** Cytokines significantly changed in SD/PD groups. CR, complete response; PR, partial response; SD, stable disease; PD, progressive disease; ns, not significant; *P<0.05, ** P<0.01.

| **Table S1. Overview of the T subset panel dedicated to a specific cell type which is indicated by individual colors.** | | | |
| --- | --- | --- | --- |
| Target | Clone | Fluorochrome | Source |
| CD3 | UCHT1 | BV395 | BD Pharmingen |
| CD4 | SK3 | BV510 | BD Pharmingen |
| CD8 | RPA-T8 | BV711 | BD Pharmingen |
| HLADR | G46-6 | PerCP-Cy5.5 | BD Pharmingen |
| CD38 | HB7 | BV605 | BD Pharmingen |
| CD25 | M-A251 | BV421 | BD Pharmingen |
| CD127 | HIL-7R-M21 | APC-R700 | BD Pharmingen |
| KI67 | B56 | AF647 | BD Pharmingen |
| GZMB | GB11 | FITC | BD Pharmingen |
| CXCR3 | IC6/CXCR3 | PE-Cy7 | BD Pharmingen |
| CCR7 | CD197 | PE | BD Pharmingen |
| CD45RA | 2D1 | APC-Cy7 | BD Pharmingen |

**Table S2. Baseline characteristics between the Camrelizumab and Sintilimab groups**

| Characteristics | Camrelizumab (n=38) | Sintilimab (n=33) | P value |
| --- | --- | --- | --- |
| Age, years, median (IQR) | 54 (48, 64) | 52 (45, 60) | 0.296 |
| Gender, n (.)  Male  Female | 36 (94.7)  2 (5.3) | 32 (97.0)  1 (3.0) | 1.000 |
| Etiology, n (.)  HBV  HCV  Others | 30 (78.9)  1 (2.6)  7 (18.4) | 30 (90.9)  0 (0)  3 (9.1) | 0.323 |
| ECOG PS, n (.)  0  1 | 35 (92.1)  3 (7.9) | 29 (87.9)  4(12.1) | 0.697 |
| Child Pugh score, n (.)  5  6 | 34 (89.5)  4 (10.5) | 29 (87.9)  4 (12.1) | 1.000 |
| ALBI score | -2.74±0.42 | -2.51±0.42 | 0.023 |
| ALBI grade, n (.)  1  2 | 23 (60.5)  15 (39.5) | 16 (48.5)  17 (51.5) | 0.437 |
| BCLC stage, n (.)  B  C | 14 (36.8)  24 (63.2) | 16 (48.5)  17(51.5) | 0.322 |
| Tumor number, n (.)  1  2  3  4 | 16 (42.1)  15 (39.5)  2 (5.3)  5 (13.1) | 13 (39.4)  13 (39.4)  3 (9.1)  4(12.1) | 0.937 |
| Largest tumor size, cm | 11.11±3.60 | 11.09±3.88 | 0.985 |
| Vascular invasion, n (.)  Both PVTT and HVTT  HVTT  PVTT  VP1/VP2/VP3/VP4 | 4 (10.5)  2(5.3)  17(44.7)  3/2/11/5 | 2 (6.1)  1 (3.0)  15(45.5)  2/4/8/3 | 0.851 |
| AFP level, n (.)  < 400 ng/mL  ≥ 400 ng/mL | 23 (60.5)  15 (39.5) | 15 (45.5)  18 (54.5) | 0.696 |

IQR, Inter quartile range; HBV, hepatitis B virus; HCV, hepatitis C virus; ECOG PS, eastern cooperative oncology group physical status; ALBI, albumin-bilirubin; BCLC, Barcelona clinic liver cancer; PVTT, portal vein tumor thrombus; HVTT, hepatic vein tumor thrombus; AFP, alpha-fetoprotein; TACE, transarterial chemoembolization; LEN-TAP, triple combination regimen of Lenvatinib, transarterial chemoembolization (TACE) and PD-1 inhibitors.

| **Table S3. Confirmed antitumor activity between the Camrelizumab and Sintilimab groups** | | | | | | | | |
| --- | --- | --- | --- | --- | --- | --- | --- | --- |
|  | | RECIST 1.1 | | |  | mRECIST | | |
| Variable | Camrelizumab | | Sintilimab | P value |  | Camrelizumab | Sintilimab | P value |
| Objective response  Complete response  Partial response | 16 (42.1)  0 (0)  16 (42.1) | | 11 (33.3)  1 (3.0)  10 (30.3) | 0.448 |  | 31 (81.6)  8 (21.1)  23 (60.5) | 25 (75.8)  5 (15.2)  20 (60.6) | 0.549 |
| Stable disease | 20 (52.6) | | 19(57.6) |  |  | 5 (13.1) | 6 (18.1) |  |
| Disease control | 36(94.7) | | 30 (90.9) | 0.658 |  | 36 (94.7) | 31 (93.9) | 1.000 |
| Progressive disease | 2 (5.3) | | 3(9.1) |  |  | 2 (5.3) | 2 (6.1) |  |
| Amenable for salvage resection | 32 | | 26 | 0.556 |  | 34 | 28 | 0.724 |
| RECIST, response evaluation criteria in solid tumors; mRECIST, modified RECIST. | | | | | | | | |

| **Table S4. Adverse events between the Camrelizumab and Sintilimab groups** | | | | | | |  |
| --- | --- | --- | --- | --- | --- | --- | --- |
| Adverse events | Any grade, n (.) | | | Grade 3, n (.) | | |  |
|  | Camrelizumab (N=38) | Sintilimab (N=33) | P | Camrelizumab (N=38) | Sintilimab (N=33) | P | |
| Abdominal pain | 24(63.6) | 18 (54.5) | 0.462 | 5 (13.2) | 5 (15.2) | 1.000 | |
| Hand-foot skin reaction | 12 (31.6) | 7 (21.2) | 0.292 | 0 (0.0) | 0 (0.0) |  | |
| Diarrhea | 15 (39.5) | 8 (24.2) | 0.171 | 3 (7.9) | 0 (0.0) | 0.243 | |
| Fatigue | 18 (47.4) | 9 (27.3) | 0.082 | 1 (2.6) | 0 (0.0) | 1.000 | |
| Nausea | 20 (52.6) | 12 (36.4) | 0.169 | 5 (13.2) | 1 (3.0) | 0.206 | |
| Vomiting | 14 (36.8) | 9 (27.3) | 0.390 | 2 (5.3) | 0 (0.0) | 0.495 | |
| Constipation | 4 (10.5) | 2 (6.1) | 0.679 | 0 (0.0) | 0 (0.0) |  | |
| Decreased appetite | 20 (52.6) | 16 (48.5) | 0.727 | 4 (10.5) | 1 (3.0) | 0.363 | |
| Rash | 10 (26.3) | 5 (15.2) | 0.250 | 0 (0.0) | 1 (3.0) | 0.465 | |
| Fever | 16 (42.1) | 8 (24.2) | 0.113 | 4 (10.5) | 0 (0.0) | 0.118 | |
| Hypertension | 16 (42.1) | 13(39.4) | 0.817 | 4 (10.5) | 2 (6.1) | 0.679 | |
| Decreased weight | 18 (47.4) | 9 (27.3) | 0.082 | 2 (5.3) | 0 (0.0) | 0.495 | |
| Proteinuria | 9 (23.6) | 6 (18.2) | 0.571 | 1 (2.6) | 0 (0.0) | 1.000 | |
| Decreased platelets | 14 (36.8) | 11 (33.3) | 0.758 | 5 (13.2) | 3 (9.1) | 0.716 | |
| Increased ALT | 23 (60.5) | 15 (45.5) | 0.309 | 5 (13.2) | 5 (15.2) | 1.000 | |
| Increased AST | 21 (55.3) | 16 (48.5) | 0.569 | 5 (13.2) | 5 (15.2) | 1.000 | |
| Hyperbilirubinemia | 6 (15.8) | 5 (15.2) | 0.941 | 0 (0.0) | 0 (0.0) |  | |
| Hypothyroidism | 8 (21.1) | 6 (18.2) | 0.762 | 0 (0.0) | 1 (3.0) | 0.465 | |
| ALT, alanine transaminase; AST, aspartate aminotransferase. | | | | | | | |

| **Table S5. Perioperative outcome between the Camrelizumab and Sintilimab groups** | | | |
| --- | --- | --- | --- |
| Variables | Camrelizumab (n=26) | Sintilimab (n=16) | P value |
| Hospital stays after surgery (days) | 8.61 ± 4.96 | 10.94 ± 4.00 | 0.122 |
| Preoperative AFP, n (.)  Normal  Elevated | 18 (69.2.)  8 (30.8.) | 7 (43.8.)  9 (56.2.) | 0.190 |
| Extent of resection, n (.)  Major  Minor | 23 (88.5.)  3 (11.5.) | 10 (62.5.)  6 (37.5.) | 0.109 |
| Operation duration (min) | 213.31 ± 71.63 | 241.38 ± 127.15 | 0.428 |
| HIO (Min) | 46.65 ± 19.77 | 37.06 ± 29.91 | 0.266 |
| Intraoperative blood loss (mL) | 500.00 ± 499.60 | 537.50 ± 387.94 | 0.787 |
| Transfusion, n (.)  Yes  No | 5 (19.2.)  21 (80.8.) | 5 (31.2.)  11 (68.8.) | 0.606 |
| Pathological response, n (.)  pCR  MPR | 9 (34.6.)  11 (42.3.) | 8 (50.0.)  2 (12.5.) | 0.126 |
| Microvascular invasion, n (.)  Present  Absent | 4 (15.4.)  22 (84.6.) | 3 (18.8.)  13 (81.2.) | 1.000 |
| Clavien-Dindo classification, n (.)  0～Ⅱ  Ⅲ～Ⅳ | 19 (73.1.)  7 (26.9.) | 13 (81.2.)  3 (18.8.) | 0.550 |
| AFP, alpha-fetoprotein; HIO, hepatic inflow occlusion; pCR, pathologic complete response; MPR, major pathologic response. | | | |

**References**

1 Eisenhauer, E. A. *et al.* New response evaluation criteria in solid tumours: revised RECIST guideline (version 1.1). *European journal of cancer (Oxford, England : 1990)*. **45**, 228-247, (2009).

2 Lencioni, R. & Llovet, J. M. Modified RECIST (mRECIST) assessment for hepatocellular carcinoma. *Seminars in liver disease*. **30**, 52-60, (2010).

3 McKenna, H. J. *et al.* Mice lacking flt3 ligand have deficient hematopoiesis affecting hematopoietic progenitor cells, dendritic cells, and natural killer cells. *Blood*. **95**, 3489-3497, (2000).

4 Waskow, C. *et al.* The receptor tyrosine kinase Flt3 is required for dendritic cell development in peripheral lymphoid tissues. *Nat Immunol*. **9**, 676-683, (2008).

5 Kimura, A. & Kishimoto, T. IL-6: regulator of Treg/Th17 balance. *Eur J Immunol*. **40**, 1830-1835, (2010).

6 McDevitt, H., Munson, S., Ettinger, R. & Wu, A. Multiple roles for tumor necrosis factor-alpha and lymphotoxin alpha/beta in immunity and autoimmunity. *Arthritis Res*. **4 Suppl 3**, S141-152, (2002).

7 Li, S. *et al.* IL-17A inhibits autophagic activity of HCC cells by inhibiting the degradation of Bcl2. *Biochem Biophys Res Commun*. **509**, 194-200, (2019).

8 Sun, D. *et al.* IL-17a promotes hepatocellular carcinoma by increasing FAP expression in hepatic stellate cells via activation of the STAT3 signaling pathway. *Cell Death Discov*. **10**, 230, (2024).

9 Jiang, W. *et al.* Insight into the role of TRAIL in liver diseases. *Biomed Pharmacother*. **110**, 641-645, (2019).

10 Kadomoto, S., Izumi, K. & Mizokami, A. The CCL20-CCR6 Axis in Cancer Progression. *Int J Mol Sci*. **21**, (2020).
