# Supplementary material for: Lenvatinib plus transarterial chemoembolization and PD-1 inhibitors as conversion therapies for unresectable intermediate-advanced hepatocellular carcinoma: a phase 2 trial and exploratory biomolecular study
Source: Signal Transduct Target Ther. 2026 Jan 22;11:37. doi: 10.1038/s41392-025-02498-z (PMC12823698; doi:10.1038/s41392-025-02498-z)
Supplement: Supplementary file 2 — Supplementary information [file 41392_2025_2498_MOESM2_ESM.docx]

**Table S1. Baseline characteristics of enrolled patients**

| Characteristics | Camrelizumab (n=38) | Sintilimab (n=33) | P value |
| --- | --- | --- | --- |
| Age, years, median (IQR) | 54 (48, 64) | 52 (45, 60) | 0.296 |
| Gender, n (.)  Male  Female | 36 (94.7)  2 (5.3) | 32 (97.0)  1 (3.0) | 1.000 |
| Etiology, n (.)  HBV  HCV  Others | 30 (78.9)  1 (2.6)  7 (18.4) | 30 (90.9)  0 (0)  3 (9.1) | 0.323 |
| ECOG PS, n (.)  0  1 | 35 (92.1)  3 (7.9) | 29 (87.9)  4(12.1) | 0.697 |
| Child Pugh score, n (.)  5  6 | 34 (89.5)  4 (10.5) | 29 (87.9)  4 (12.1) | 1.000 |
| ALBI score | -2.74±0.42 | -2.51±0.42 | 0.023 |
| ALBI grade, n (.)  1  2 | 23 (60.5)  15 (39.5) | 16 (48.5)  17 (51.5) | 0.437 |
| BCLC stage, n (.)  B  C | 14 (36.8)  24 (63.2) | 16 (48.5)  17(51.5) | 0.322 |
| Tumor number, n (.)  1  2  3  4 | 16 (42.1)  15 (39.5)  2 (5.3)  5 (13.1) | 13 (39.4)  13 (39.4)  3 (9.1)  4(12.1) | 0.937 |
| Largest tumor size, cm | 11.11±3.60 | 11.09±3.88 | 0.985 |
| Vascular invasion, n (.)  Both PVTT and HVTT  HVTT  PVTT  VP1/VP2/VP3/VP4 | 4 (10.5)  2(5.3)  17(44.7)  3/2/11/5 | 2 (6.1)  1 (3.0)  15(45.5)  2/4/8/3 | 0.851 |
| AFP level, n (.)  < 400 ng/mL  ≥ 400 ng/mL | 23 (60.5)  15 (39.5) | 15 (45.5)  18 (54.5) | 0.696 |

IQR, Inter quartile range; HBV, hepatitis B virus; HCV, hepatitis C virus; ECOG PS, eastern cooperative oncology group physical status; ALBI, albumin-bilirubin; BCLC, Barcelona clinic liver cancer; PVTT, portal vein tumor thrombus; HVTT, hepatic vein tumor thrombus; AFP, alpha-fetoprotein; TACE, transarterial chemoembolization; LEN-TAP, triple combination regimen of Lenvatinib, transarterial chemoembolization (TACE) and PD-1 inhibitors.

| **Table S2. Confirmed antitumor activity after conversion therapy** | | | | | | | | |
| --- | --- | --- | --- | --- | --- | --- | --- | --- |
|  | | RECIST 1.1 | | |  | mRECIST | | |
| Variable | Camrelizumab | | Sintilimab | P value |  | Camrelizumab | Sintilimab | P value |
| Objective response  Complete response  Partial response | 16 (42.1)  0 (0)  16 (42.1) | | 11 (33.3)  1 (3.0)  10 (30.3) | 0.448 |  | 31 (81.6)  8 (21.1)  23 (60.5) | 25 (75.8)  5 (15.2)  20 (60.6) | 0.549 |
| Stable disease | 20 (52.6) | | 19(57.6) |  |  | 5 (13.1) | 6 (18.1) |  |
| Disease control | 36(94.7) | | 30 (90.9) | 0.658 |  | 36 (94.7) | 31 (93.9) | 1.000 |
| Progressive disease | 2 (5.3) | | 3(9.1) |  |  | 2 (5.3) | 2 (6.1) |  |
| Amenable for salvage resection | 32 | | 26 | 0.556 |  | 34 | 28 | 0.724 |
| RECIST, response evaluation criteria in solid tumors; mRECIST, modified RECIST. | | | | | | | | |

| **Table S3. Adverse events after conversion therapy** | | | | | | |  |
| --- | --- | --- | --- | --- | --- | --- | --- |
| Adverse events | Any grade, n (.) | | | Grade 3, n (.) | | |  |
|  | Camrelizumab (N=38) | Sintilimab (N=33) | P | Camrelizumab (N=38) | Sintilimab (N=33) | P | |
| Abdominal pain | 24(63.6) | 18 (54.5) | 0.462 | 5 (13.2) | 5 (15.2) | 1.000 | |
| Hand-foot skin reaction | 12 (31.6) | 7 (21.2) | 0.292 | 0 (0.0) | 0 (0.0) |  | |
| Diarrhea | 15 (39.5) | 8 (24.2) | 0.171 | 3 (7.9) | 0 (0.0) | 0.243 | |
| Fatigue | 18 (47.4) | 9 (27.3) | 0.082 | 1 (2.6) | 0 (0.0) | 1.000 | |
| Nausea | 20 (52.6) | 12 (36.4) | 0.169 | 5 (13.2) | 1 (3.0) | 0.206 | |
| Vomiting | 14 (36.8) | 9 (27.3) | 0.390 | 2 (5.3) | 0 (0.0) | 0.495 | |
| Constipation | 4 (10.5) | 2 (6.1) | 0.679 | 0 (0.0) | 0 (0.0) |  | |
| Decreased appetite | 20 (52.6) | 16 (48.5) | 0.727 | 4 (10.5) | 1 (3.0) | 0.363 | |
| Rash | 10 (26.3) | 5 (15.2) | 0.250 | 0 (0.0) | 1 (3.0) | 0.465 | |
| Fever | 16 (42.1) | 8 (24.2) | 0.113 | 4 (10.5) | 0 (0.0) | 0.118 | |
| Hypertension | 16 (42.1) | 13(39.4) | 0.817 | 4 (10.5) | 2 (6.1) | 0.679 | |
| Decreased weight | 18 (47.4) | 9 (27.3) | 0.082 | 2 (5.3) | 0 (0.0) | 0.495 | |
| Proteinuria | 9 (23.6) | 6 (18.2) | 0.571 | 1 (2.6) | 0 (0.0) | 1.000 | |
| Decreased platelets | 14 (36.8) | 11 (33.3) | 0.758 | 5 (13.2) | 3 (9.1) | 0.716 | |
| Increased ALT | 23 (60.5) | 15 (45.5) | 0.309 | 5 (13.2) | 5 (15.2) | 1.000 | |
| Increased AST | 21 (55.3) | 16 (48.5) | 0.569 | 5 (13.2) | 5 (15.2) | 1.000 | |
| Hyperbilirubinemia | 6 (15.8) | 5 (15.2) | 0.941 | 0 (0.0) | 0 (0.0) |  | |
| Hypothyroidism | 8 (21.1) | 6 (18.2) | 0.762 | 0 (0.0) | 1 (3.0) | 0.465 | |
| ALT, alanine transaminase; AST, aspartate aminotransferase. | | | | | | | |

| **Table S4. Perioperative outcome for patients underwent salvage liver resection** | | | |
| --- | --- | --- | --- |
| Variables | Camrelizumab (n=26) | Sintilimab (n=16) | P value |
| Hospital stays after surgery (days) | 8.61 ± 4.96 | 10.94 ± 4.00 | 0.122 |
| Preoperative AFP, n (.)  Normal  Elevated | 18 (69.2.)  8 (30.8.) | 7 (43.8.)  9 (56.2.) | 0.190 |
| Extent of resection, n (.)  Major  Minor | 23 (88.5.)  3 (11.5.) | 10 (62.5.)  6 (37.5.) | 0.109 |
| Operation duration (min) | 213.31 ± 71.63 | 241.38 ± 127.15 | 0.428 |
| HIO (Min) | 46.65 ± 19.77 | 37.06 ± 29.91 | 0.266 |
| Intraoperative blood loss (mL) | 500.00 ± 499.60 | 537.50 ± 387.94 | 0.787 |
| Transfusion, n (.)  Yes  No | 5 (19.2.)  21 (80.8.) | 5 (31.2.)  11 (68.8.) | 0.606 |
| Pathological response, n (.)  pCR  MPR | 9 (34.6.)  11 (42.3.) | 8 (50.0.)  2 (12.5.) | 0.126 |
| Microvascular invasion, n (.)  Present  Absent | 4 (15.4.)  22 (84.6.) | 3 (18.8.)  13 (81.2.) | 1.000 |
| Clavien-Dindo classification, n (.)  0～Ⅱ  Ⅲ～Ⅳ | 19 (73.1.)  7 (26.9.) | 13 (81.2.)  3 (18.8.) | 0.550 |
| AFP, alpha-fetoprotein; HIO, hepatic inflow occlusion; pCR, pathologic complete response; MPR, major pathologic response. | | | |
