# Supplementary figures and images for: Lenvatinib plus transarterial chemoembolization and PD-1 inhibitors as conversion therapies for unresectable intermediate-advanced hepatocellular carcinoma: a phase 2 trial and exploratory biomolecular study
Source: Signal Transduct Target Ther. 2026 Jan 22;11:37. doi: 10.1038/s41392-025-02498-z (PMC12823698; doi:10.1038/s41392-025-02498-z)

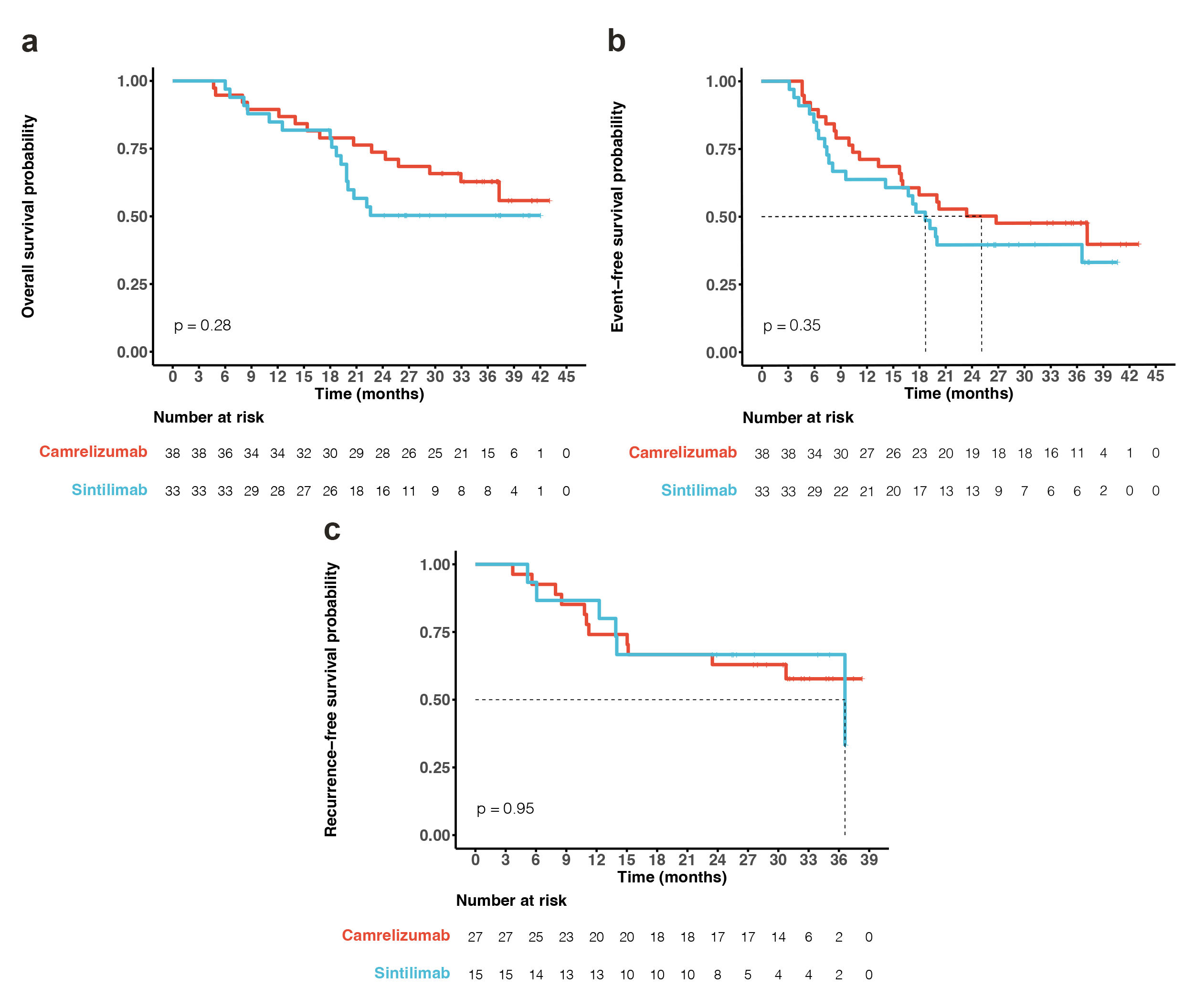

Supplement: Supplementary file 3 — SIGTRANS-15441-s03 [file 41392_2025_2498_MOESM3_ESM.tif]

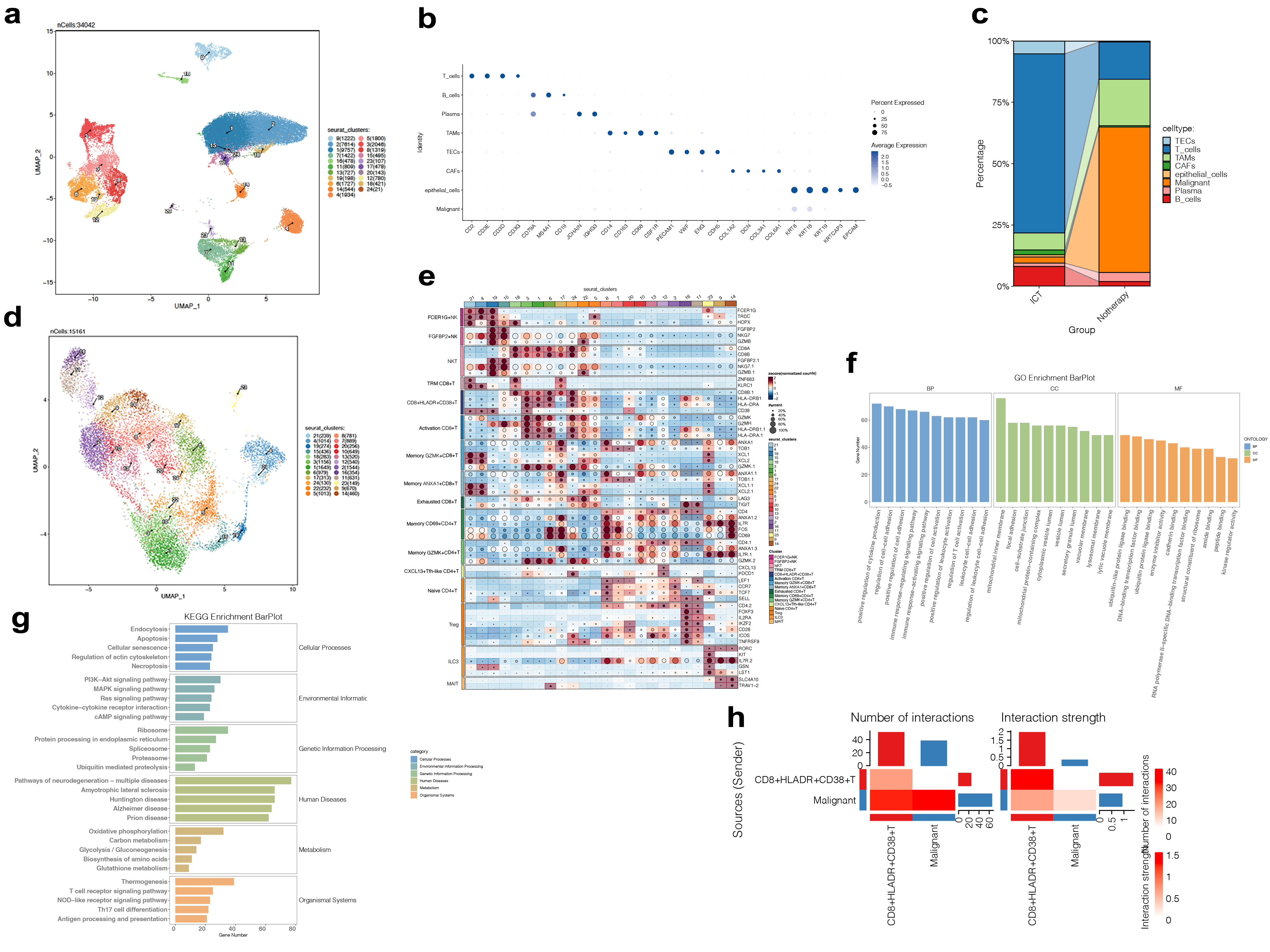

Supplement: Supplementary file 5 — SIGTRANS-15441-s05 [file 41392_2025_2498_MOESM5_ESM.tif]

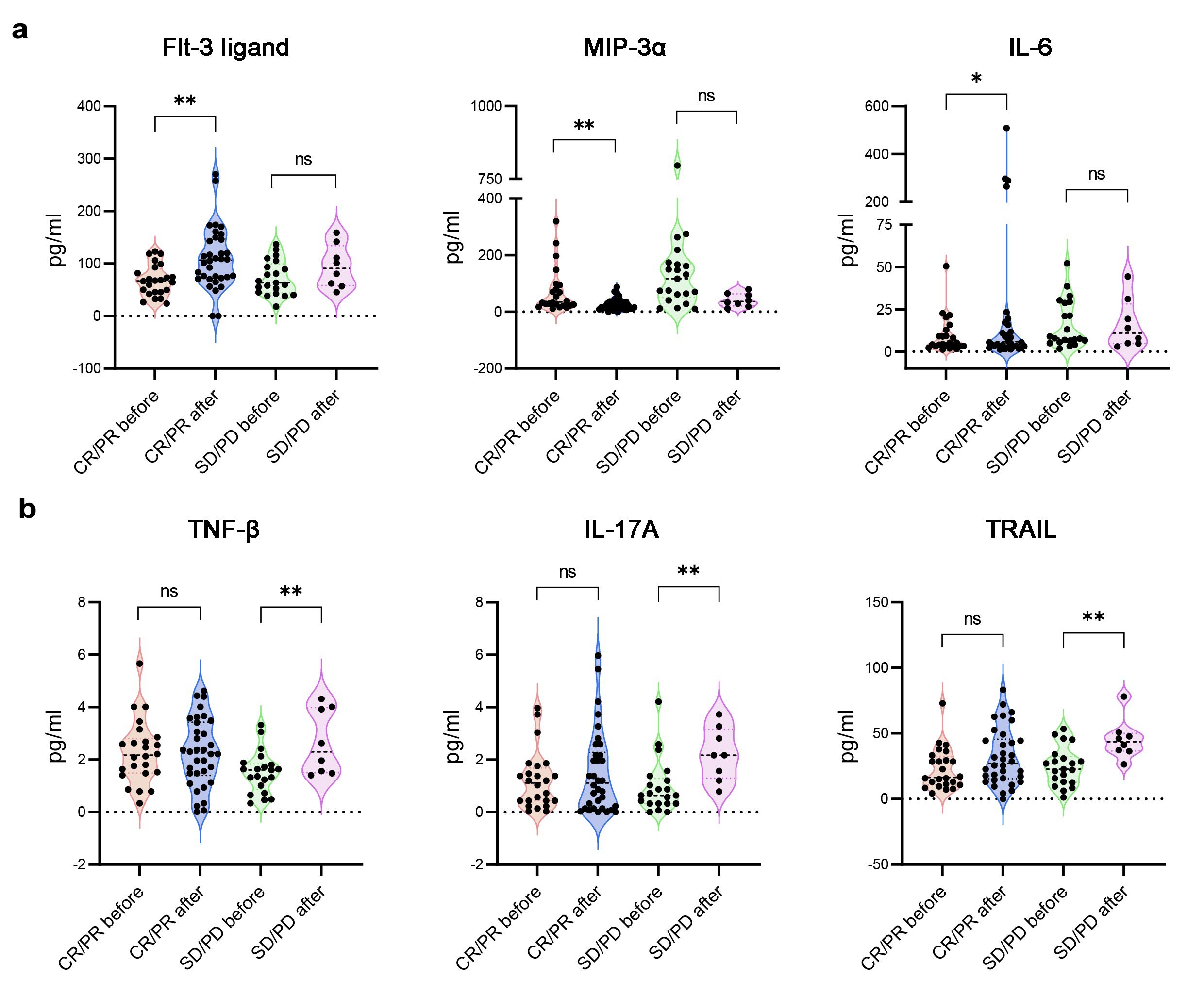

Supplement: Supplementary file 6 — SIGTRANS-15441-s06 [file 41392_2025_2498_MOESM6_ESM.tif]
